# A Specificity Map for the PDZ Domain Family

**DOI:** 10.1371/journal.pbio.0060239

**Published:** 2008-09-30

**Authors:** Raffi Tonikian, Yingnan Zhang, Stephen L Sazinsky, Bridget Currell, Jung-Hua Yeh, Boris Reva, Heike A Held, Brent A Appleton, Marie Evangelista, Yan Wu, Xiaofeng Xin, Andrew C Chan, Somasekar Seshagiri, Laurence A Lasky, Chris Sander, Charles Boone, Gary D Bader, Sachdev S Sidhu

**Affiliations:** 1 Terrence Donnelly Center for Cellular and Biomolecular Research, Banting and Best Department of Medical Research, University of Toronto, Toronto, Ontario, Canada; 2 Department of Molecular Genetics, University of Toronto, Toronto, Ontario, Canada; 3 Department of Protein Engineering, Genentech, South San Francisco, California, United States of America; 4 Department of Biological Engineering, Massachusetts Institute of Technology, Cambridge, Massachusetts, United States of America; 5 Department of Molecular Biology, Genentech, South San Francisco, California, United States of America; 6 Department of Immunology, Genentech South San Francisco, California, United States of America; 7 Computational Biology Center, Memorial Sloan-Kettering Cancer Center, New York, New York, United States of America; 8 Department of Antibody Engineering, Genentech, South San Francisco, California, United States of America; Howard Hughes Medical Institute, Janelia Farm, United States of America

## Abstract

PDZ domains are protein–protein interaction modules that recognize specific C-terminal sequences to assemble protein complexes in multicellular organisms. By scanning billions of random peptides, we accurately map binding specificity for approximately half of the over 330 PDZ domains in the human and Caenorhabditis elegans proteomes. The domains recognize features of the last seven ligand positions, and we find 16 distinct specificity classes conserved from worm to human, significantly extending the canonical two-class system based on position −2. Thus, most PDZ domains are not promiscuous, but rather are fine-tuned for specific interactions. Specificity profiling of 91 point mutants of a model PDZ domain reveals that the binding site is highly robust, as all mutants were able to recognize C-terminal peptides. However, many mutations altered specificity for ligand positions both close and far from the mutated position, suggesting that binding specificity can evolve rapidly under mutational pressure. Our specificity map enables the prediction and prioritization of natural protein interactions, which can be used to guide PDZ domain cell biology experiments. Using this approach, we predicted and validated several viral ligands for the PDZ domains of the SCRIB polarity protein. These findings indicate that many viruses produce PDZ ligands that disrupt host protein complexes for their own benefit, and that highly pathogenic strains target PDZ domains involved in cell polarity and growth.

## Author Summary

The PDZ domain is a structural domain that functions as a protein–protein interaction module that recognizes specific C-terminal peptide sequences to assemble intracellular complexes important in signaling pathways of multicellular organisms. These modules are associated with human disease and are targets of viruses and other pathogens. By examining peptide specificity and substrate diversity of roughly one half of the PDZ domains known to exist in human and the nematode Caenorhabditis elegans, we were able to show that PDZ domains are more specific than previously appreciated. PDZ domains also remain functional under high mutational pressure, and only a few of the vast number of possible PDZ domain specificities are utilized in nature. These PDZ domain specificities are conserved from human to worm, implying that the specificities evolved early and were reused over evolution instead of being reshaped. The specificity map generated here was used to predict and experimentally confirm new viral PDZ-binding motifs. We present evidence that pathogenic viruses, including avian influenza, bind host PDZ domains via these motifs, thereby competing with signaling by host complexes, which leads to disruption of growth and polarity of the host cells.

## Abstract 3

A genome-scale specificity map for PDZ domains reveals how family members recognize ligands to assemble signaling complexes and also reveals how viruses target these domains to subvert host cell function.

## Introduction

Modular protein–protein recognition domains are involved in the assembly of numerous intracellular complexes that mediate diverse cellular functions. Thousands of recognition domains are contained within the human proteome, and these have been classified into over 70 distinct families [1]. The PDZ (PSD-95/Discs-large/ZO-1) domain family is particularly interesting because it plays a key role in the development of multicellular organisms, in which PDZ domains are often found as components of multidomain scaffolding proteins involved in cell polarity and intercellular interactions [2,3]. PDZ domains are often embedded in proteins that assemble specialized subcellular sites, such as epithelial junctions [4], neuronal postsynaptic densities [5], and immunological synapses of T cells [6]. The biological importance of PDZ domains is further underscored by the identification of various PDZ-containing proteins as human disease and pathogen effector targets [4,7–15].

Although the human genome encodes over 250 PDZ domains in over 100 proteins, most studies to date have focused on individual family members or a handful of domains [16–20]. Nevertheless, these studies uncovered general features of PDZ domain structure and function. Aside from unusual cases in which phospholipids [21,22] or internal motifs [23,24] are recognized, PDZ domains assemble intracellular complexes principally by recognition of C-terminal sequences in which specificity is mediated by interactions between ligand side chains and the PDZ domain binding surface [2]. Early studies grouped PDZ domains into two main specificity classes based on two ligand positions: class 1 (*X*[T/S]*X*φ_COOH_) and class 2 (*X*φ*X*φ_COOH_), where *X* is any residue and φ is a hydrophobe [16,25]. Less common classes of PDZ domains, such as class 3 recognizing the motif *X*[ED]*X*φ_COOH_, were also identified [17]. However, subsequent studies have shown that the PDZ binding cleft can interact specifically with up to seven C-terminal ligand residues, enabling differentiation between biologically diverse ligands [20]. A recent large-scale analysis of mouse PDZ domains confirmed the highly specific nature of PDZ–ligand interactions but did not address the issue of PDZ domain classification [26]. To better understand how PDZ domains mediate cellular function and how hundreds of family members may compete for hundreds of potential ligands, we conducted a large-scale analysis using phage-displayed random peptide libraries. This establishes a specificity map and comprehensive classification system for the PDZ domain family, which provides insight into domain function and evolution and can be used to make novel discoveries about PDZ domain signaling systems.

## Results

### PDZ Domain Specificity Potential

We used C-terminal peptide-phage display [27,28] to conduct a large-scale analysis of PDZ domain specificity, focusing on human and, for comparison, the simple metazoan Caenorhabditis elegans. Previous studies have shown that a phage-displayed combinatorial peptide library approach is a powerful tool to elucidate PDZ domain specificity and may be used to identify biologically relevant targets [20,29,30]. We cloned 72 out of 82 worm and 96 out of 254 human PDZ domains detected by three domain detection tools, BLAST [31], PFAM [32], and SMART [33]. The domain boundaries were defined as the union of all predicted domain regions plus ten amino acids on each side.

The 168 cloned domains were expressed as GST fusion proteins, and 145 of these (57 worm and 88 human) could be purified in a stable, soluble form. These 145 proteins were used in binding selections with a C-terminal phage-displayed library containing greater than 10 billion random peptides, and we were successful in obtaining binding peptides against 82 domains (28 worm and 54 human). Failure to find binding peptides for the remaining domains may be due to instability of isolated domains or a requirement for larger, structured ligands not represented in our random peptide library [2]. Nonetheless, by sequencing approximately 10,000 binding clones, we were able to isolate approximately 3,100 unique peptide ligands for 82 PDZ domains (Tables S1 and S2). The domains used in this analysis and all associated peptides are available in a computer-readable format at http://baderlab.org/Data/PDZ.

**Figure 1:**
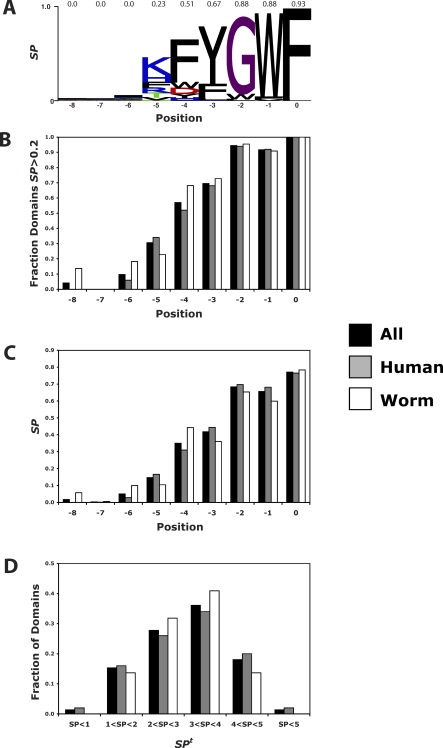
PDZ Domains Are Highly Specific across Multiple Ligand Positions A total of 72 PDZ domains (each with greater than ten peptides) corresponding to 2,998 ligands were analyzed to assess specificity for each ligand position. Specificity was measured using the *SP* score, which ranges from zero (least specific) at a given ligand position to one (most specific). Bars are colored as follows: all PDZ domains (black), human (grey), or worm (white). (A) Specificity profile for a representative PDZ domain (C34F11.9a-1) with *SP* scores shown above each ligand position. (B) Fraction of PDZ domains exhibiting significant specificity (*SP* > 0.2) at each ligand position. (C) The mean *SP* value at each ligand position. (D) The distribution of total *SP* (*SP ^t^*) summed over all ligand positions.

Consistent with the canonical preference of PDZ domains for hydrophobic C termini, the vast majority of the selected sequences (>97%) terminate with a hydrophobic residue, and for each domain, we therefore aligned the sequences on the basis of the C-terminal anchor position. The small number of peptides that are not canonical C-terminal binders were not considered in our analysis, although they are made available in our peptide files for others to analyze. To statistically model the binding specificity of each PDZ domain to enable computational analysis, each aligned peptide ligand set was used to create a position weight matrix (PWM). Each matrix column captures the amino acid binding preference of a PDZ domain at a ligand position as a probability distribution. From this PWM, the specificity of each ligand position is visualized as a sequence logo [34] and summarized using a specificity potential (*SP*) score ranging from least specific (any amino acid is recognized, *SP* score = zero) to most specific (only a single amino acid is recognized, *SP* score = one) (Figure 1A). For 72 PDZ domains, we had sufficient peptide data (*n* > 10) to calculate reliable *SP* scores. Our analysis reveals that essentially all PDZ domains recognize the last three ligand positions (0, −1, and −2), the majority recognize positions −3 and −4, and some recognize positions −5 and −6 (Figure 1B, *SP* > 0.2; Figure 1C, mean *SP*). The total specificity score per domain (*SP ^t^*), calculated by summing the *SP* scores across the last nine ligand positions (Figure 1D), shows that most PDZ domain binding sites achieve high specificity through recognition of multiple features of the last seven residues of C-terminal peptide ligands (Table S3). These patterns are conserved across worm and human. Furthermore, there is no significant difference between the *SP ^t^* values for worm (mean = 3.2 ± 0.9) and human domains (mean = 3.1 ± 1.0), indicating that increased human genome complexity has not been accompanied by a corresponding increase in overall PDZ domain specificity.

**Figure 2:**
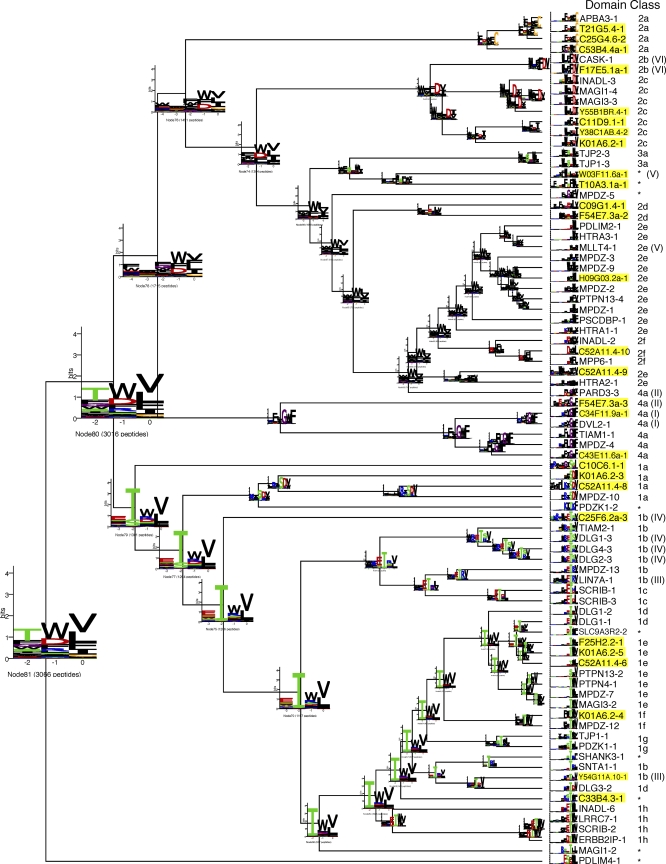
Specificity Map Classifies the PDZ Domain Family All 82 PDZ domains studied were clustered to create a specificity map, which was used as a guide to manually define PDZ specificity classes. Of the 82 domains, 73 are assigned to one of 16 classes, labeled to the right of each domain name. For consistency with the established PDZ domain classification system [16], each class is denoted by a numeral based on the specificity for position −2, followed by a letter to account for specificity across the rest of the binding site. C. elegans domains are highlighted in yellow. Sets marked with identical Roman numerals in parentheses are homologous PDZ domains in human/worm orthologs. Domains that exhibit unique specificities not part of any class are denoted by asterisks (*). The 16 classes are defined by the following C-terminal motifs: 1a (φ[K/R]*X*SDV); 1b (Ω[R/K]ET[S/T/R/K]φ); 1c (φφET*X*L); 1d (ET*X*V); 1e (TWΨ); 1f (ΩΩTWΨ); 1g (φφφ[T/S][T/S]ΩΨ); 1h (φφ[D/E][T/S]WΨ); 2a (FDΩΩC); 2b (W*X*ΩFDV); 2c (WΩφDΨ); 2d (φφ*X*[E/D]φφφ); 2e (φφφφ); 2f ([D/E]φΩφ); 3a (WΩ[S/T]DWΨ); 4a (*Ω*φGWF); φ, hydrophobic (V, I, L, F, W, Y, M); Ω, aromatic (F, W, and Y); Ψ, aliphatic (V, I, L, and M); and *X*, nonspecific.

We created a specificity map organizing all 82 successfully mapped PDZ domains. Hierarchical clustering was used to automatically place similar PDZ binding profiles (described by PWMs) in close proximity (Figure 2). This map reveals that approximately 90% of the domains fit into 16 distinct specificity classes, and the remainder represent unique specificities. By considering all recognized ligand positions, our comprehensive specificity map significantly expands the canonical PDZ domain classification system, which assigned only two main classes on the basis of specificity for ligand position −2 [16].

### PDZ Domains Are Versatile and Robust

**Figure 3:**
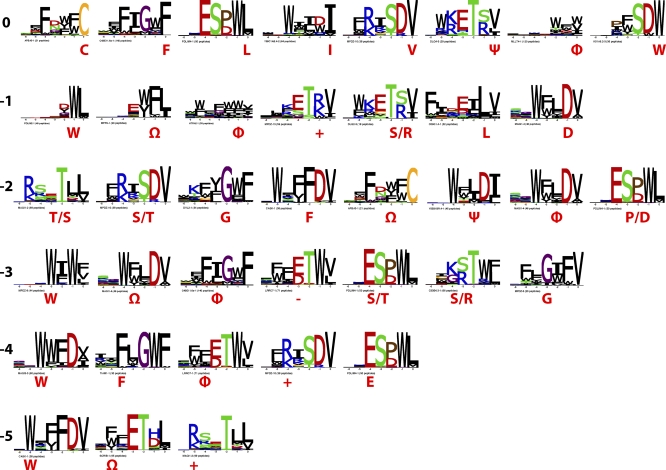
Distinct Specificities of PDZ Domain Binding Sites The specificity profiles of 72 PDZ domains reveal eight, seven, eight, seven, five, and three distinct specificities for ligand positions 0, −1, −2, −3, −4, and −5, respectively. At each position, distinct specificities are shown (magenta) with either the single-letter amino acid code or symbols, as follows: +, positive charge; *−*, negative charge; φ, hydrophobic (V, I, L, F, W, Y, and M); Ψ, aliphatic (V, I, L, and M); and Ω, aromatic (F, W, and Y).

Considering each ligand position independently, there are a striking number of distinct specificities for the last six positions (Figure 3). For instance, all domains prefer hydrophobic C termini, but there are eight distinct specificities for position 0. This suggests a vast potential for the PDZ domain family to bind different sequences. To assess the contributions of domain binding site positions to ligand binding and the specificity capacity of the PDZ domain family, we mutated ten Erbin PDZ domain (ERBB2IP-1) core binding-site positions and determined specificity profiles for each of the 91 single-residue mutants. The core binding positions are those that make contact (closer than 4.5 Å) with the peptide ligand in all of nine different PDZ domain structures (Protein Data Bank [PDB] entries 1N7T, 2H2B, 2H2C, 1I92, 2HE2, 1BE9, 2GZV, 1IHJ, and 1N7F). At each of the ten positions, mutations were made to amino acids that are abundant in the 82 natural human and worm PDZ domains for which we collected phage-derived specificity profiles (Figure 2).

**Figure 4:**
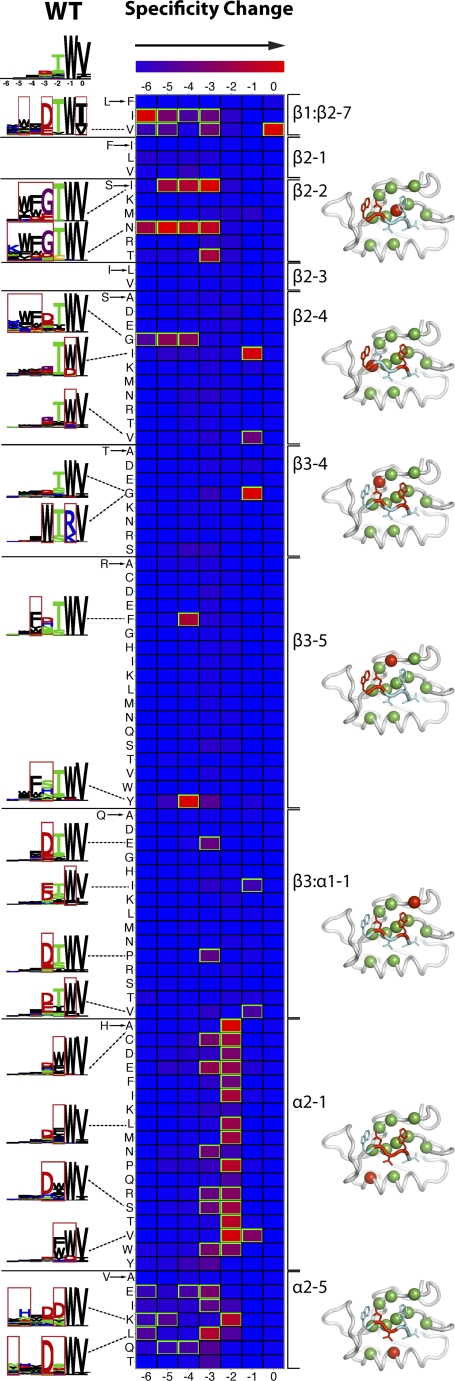
Sequence Determinants of PDZ Domain Specificity Heat map summary of the effects of mutations on the specificity of ERBB2IP-1. Each row represents one mutant, ordered by PDZ domain binding-site position (labeled to the right of each set of rows), and each column represents one ligand position. Mutations were chosen to represent the diversity of amino acids in 82 natural PDZ domains for which we have phage data. To minimize potential destabilization caused by structurally deleterious mutations, selections were performed at 4 °C, and under these low stringency conditions, the wild-type specificity profile, shown at top left, was somewhat less specific than that at room temperature (Figure 2). The mutation listed to the left of each row, at the PDZ domain position listed at the right according to a structure-based nomenclature [55], causes a change in specificity, shown in each row. The blue-to-red gradient indicates increasing difference relative to wild type, normalized per column with significant differences highlighted in green (greater than one standard deviation away from the mean difference over the column). Selected mutant profiles are highlighted (depicted as sequence logos to the left of the corresponding row), with significant specificity changes in the logo boxed in red. Structures of ERBB2IP-1 with a bound peptide ligand [36] are shown with mutated positions depicted as spheres. Red side chains indicate ligand positions for which specificity is altered by mutations at PDZ positions shown as red spheres.

To minimize potential destabilization caused by structurally deleterious mutations, selections were performed at 4 °C, and under these low stringency conditions, the wild-type specificity profile (Figure 4) was somewhat less specific than that at room temperature (wild-type ERBB2IP-1 logo in Figure 2, class 1h). Phage selections were successful in all cases, and a total of approximately 3,400 unique ligands were sequenced. We compared the specificity profile of each mutant (Figure S1) to that of the wild type and visualized the differences as a heat map (Figure 4).

Positions 0 and −2 define the most commonly used PDZ classification system [16]. The only significant change in specificity for position 0 was due to a substitution at position β1:β2–7, which lines the hydrophobic pocket that accepts the C-terminal ligand side chain. Changes in specificity for position −2 were caused by mutations in the α2 helix positions α2–1 and α2–5, which are close to the ligand side chain at this position [35]. The four substitutions at position α2–1 that do not alter specificity significantly (Y, N, Q, and K) are all capable of forming hydrogen bonds, and thus, can substitute functionally for the wild-type H, which hydrogen bonds with T at position −2 of an optimal ligand for ERBB2IP-1 [36]. The remaining 14 mutations result in class 2 specificity profiles with preference for hydrophobes at position −2. These results indicate that specificity for ligand positions −2 and 0 depends mainly on direct amino acid residue side chain interactions.

Wild-type ERBB2IP-1 prefers W at ligand position −1, and although no mutation completely alters this preference, six mutations expand specificity to include other residue types. These mutations occur at four positions spread throughout the PDZ domain, and only the effects at one position (β3:α1–1) can be explained by changes in direct residue contacts. Mutations at two positions located far from position −1 (α2–1 and β2–4) expand specificity to include D, and it is likely that these mutations alter the ligand orientation and allow a D side chain to interact with the R side chain at position β3–5, which sits between ligand positions −1 and −3 [36]. The influence of indirect effects is demonstrated dramatically by the introduction of a flexible G residue at β3–4, which produces a PDZ domain with two distinct specificity profiles, one wild type and the other completely altered at positions −1 and −3. Thus, specificity for ligand position −1 can be influenced by direct and indirect interactions at positions throughout the PDZ binding site.

Mutations at seven PDZ positions affect specificity for position −3, but only three of these positions (β2–2, β3–4, and β3–5) are in direct contact with this ligand position. Interestingly, many mutations at four other positions (β1:β2–7, β3:α1–1, α2–1, and α2–5) accentuate, rather than alter, the wild-type preference for negatively charged residues at position −3. A similar situation appears to exist for the upstream −4 and −5 positions, as the slight preference of the wild-type domain for hydrophobic residues at these positions (Figure 2, class 1h) is accentuated by mutations at five positions (β1:β2–7, β2–2, β2–4, β3–5, and α2–5), and most of these effects cannot be explained by changes in direct contacts. These effects may be caused by ligand orientation changes, which may allow for more favorable interactions between the PDZ domain and ligand residues upstream of position −2. Additionally, some mutations may weaken the energetic contributions from interactions with the three C-terminal ligand positions, and thus, ligand binding may become more dependent on favorable interactions involving upstream ligand positions. Thus, weaker interactions with ligand residues at positions −3, −4, and −5 can be affected in multiple indirect ways by mutations at numerous positions in the binding site.

**Figure 5:**
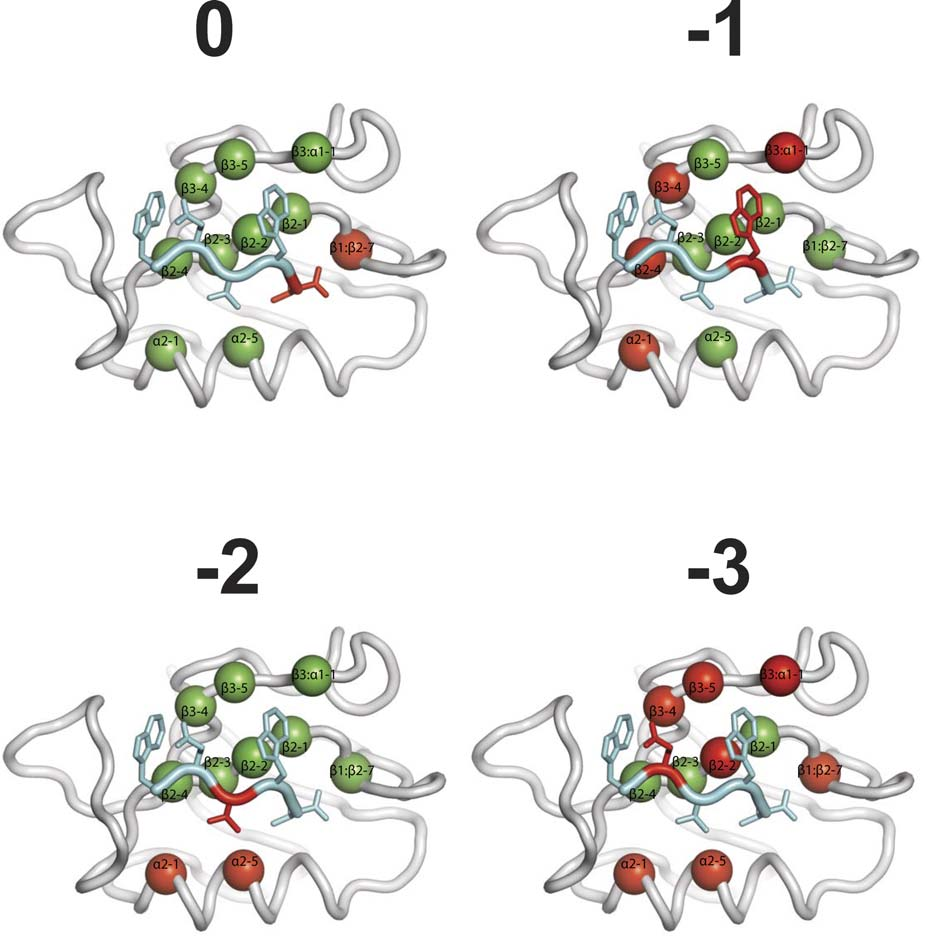
Mutations Affecting PDZ Domain Specificity ERBB2IP-1 (grey) is shown with a bound peptide ligand (WETWV_COOH_; cyan) (PDB entry 1N7T) [36]. PDZ domain binding-site positions that were subjected to mutagenesis are shown as spheres. In each panel, PDZ domain positions at which mutations affected specificity for the indicated ligand position are colored red and other mutagenized positions are colored green. PDZ domain positions are labeled in black according to a structure-based nomenclature [55].

Our mutational and specificity prediction analyses provide general insights into PDZ domain specificity and have implications for prediction of binding specificity from domain sequence. Because specificity for ligand position −2 is mediated by local contacts with the PDZ α2 helix, point mutations at α2–1 and α2–5 are sufficient to substantially alter this specificity. Furthermore, there is a strong correlation between specificity for ligand position −2 and the sequence at the α2–1 position, as 44 of our 82 mapped domains (Figure 2) contain an H at the α2–1 position, and 37 (84%) of these prefer ligands containing T/S at position −2. We were not able to detect other strong correlations between individual PDZ domain positions and ligand specificity. In contrast, specificity for −1, −3, and positions further upstream depends on positions scattered throughout the PDZ domain and likely involves indirect conformational effects that subtly alter specificity at these positions without changing the specificity class. Thus, specificity in a PDZ domain is determined by multiple structural and chemical mechanisms involving both direct contacts and cooperative, long-range effects (Figure 5). Consequently, the binding site must be considered as a whole to accurately predict specificity from primary sequence.

The clear selection of ligands by all PDZ mutants shows that the domain can function under high mutational pressure. Furthermore, 35 of 91 mutations analyzed caused a significant change in specificity for at least one ligand position (Figure 4). Taken together, these results show that PDZ domains are versatile and robust, as mutations frequently cause a change, rather than a loss of function.

### Conserved Specificity and Domain Expansion

**Figure 6:**
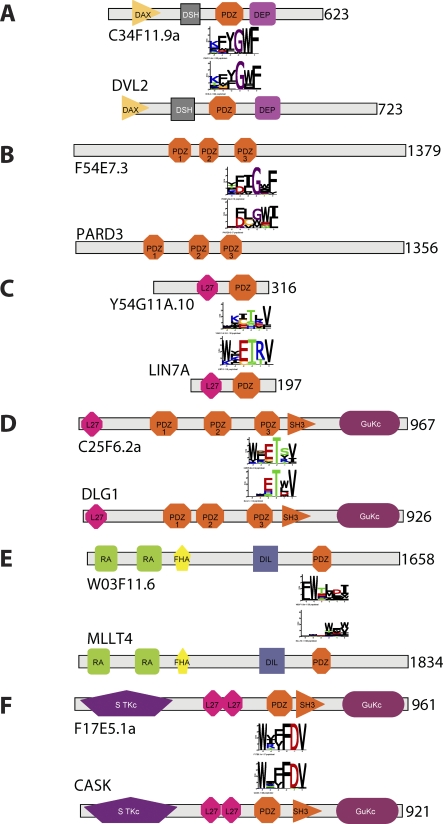
Specificity Profiles of Orthologous Domains Are Highly Conserved All worm and human ortholog pairs with mapped PDZ domains in our dataset are shown. The domain architecture, as defined by SMART [33], is shown for each worm (top) and human (bottom) protein in an ortholog pair. The specificity profiles defined by peptide phage display are shown below or above the worm or human PDZ domains, respectively. The name and length of each protein is indicated on the left or right, respectively. The orthologous protein pairs are drawn to scale. The following protein pairs could be unambiguously identified as orthologs on the basis of common domain architecture and high sequence identity: (A) C34F11.9a/DVL2, (B) F54E7.3/PARD3, (C) Y54G11A.10/LIN7A, (D) C25F6.2a/DLG1, (E) W03F11.6/MLLT4, and (F) F17E5.1a/CASK.

Because expansion of PDZ and other modular domains is correlated with increased organism complexity [37], we asked what role PDZ versatility plays in the evolution of complexity. Almost all PDZ specificity classes we define contain human and worm representatives, indicating that most of the human specificities are also present in the worm. Our dataset contains six worm/human ortholog gene pairs with mapped PDZ domain binding specificity in both species (Figure 6). Four of these pairs have nearly identical specificity profiles, and two are very similar. This level of conservation across more than one billion years of evolution separating worm from human [38] suggests that these specificity profiles are important for biological function. The limited number of conserved specificity classes used across two distant species suggests that most PDZ domain specificity classes arose early in evolution, and evolutionary constraints prevented new classes from forming following the divergence of worm and human. Consequently, additional complexity in the human PDZ domain family compared to that of the worm apparently arose through domain expansion and shuffling, rather than from the evolution of radical new specificities.

### Specificity Predicted from Primary Sequence

**Figure 7:**
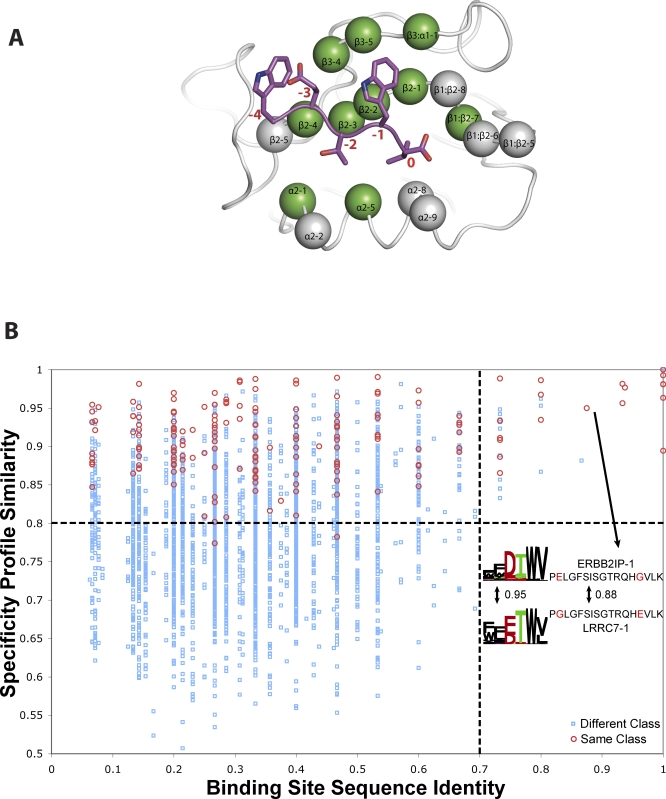
PDZ Domain Sequence Identity Accurately Predicts Binding Specificity (A) ERBB2IP-1 structure (grey) is shown with a bound peptide ligand (WETWV_COOH_; colored) [36]. PDZ domain binding site positions are shown as spheres, and positions that were analyzed by mutagenesis are colored green. PDZ positions are labeled in black according to a structure-based nomenclature [55], and peptide positions are labeled in red. We defined the PDZ binding site as 17 residues that make contact with the ligand (closer than 4.5 Å) in at least one of nine different structures (PDB entries 1N7T, 2H2B, 2H2C, 1I92, 2HE2, 1BE9, 2GZV, 1IHJ, and 1N7F). (B) The relationship between binding-site sequence identity and specificity profile similarity. Each point represents a pair of PDZ domains from our mapped set. Red circles represent pairs assigned to the same class, as defined in our specificity map, and blue squares represent all other pairs. The lower-right quadrant, absent of data points, contains an example for one pair of PDZ domains (ERBB2IP-1 and LRRC7–1), which exhibit a specificity profile similarity of 0.95 and a binding-site sequence identity of 0.88 (sequence mismatches are shown in red).

**Figure 8:**
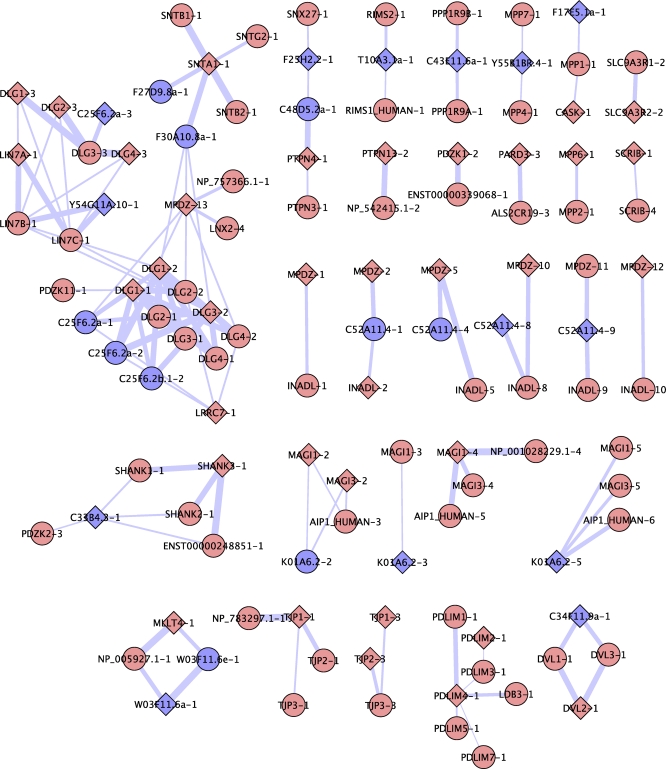
Prediction of PDZ Domain Specificity A network view of predicted PDZ domain specificities. Worm and human PDZ domains are shown as blue or pink nodes, respectively. Diamonds denote domains with experimentally phage-mapped specificity profiles, and circles denote domains with predicted specificity profiles. Lines connect domains with greater than 70% sequence identity in the binding site, and line width is proportional to sequence identity. Connected domains are predicted to have high specificity profile similarity scores (>0.83). Network was created using Cytoscape 2.5 [54].

Given the limited number of natural specificity classes and the mutant Erbin PDZ domain–ligand correlations observed, we asked whether we could use primary sequence to classify binding specificity of wild-type PDZ domains. Our extensive dataset based on phage-displayed random peptide libraries shows a clear correlation between binding-site identity and specificity. Domain pairs with binding-site sequence identities greater than 70% have specificity profiles with equivalent similarity to those within a specificity map class (Figure 7). This is the first time such a correlation has been shown for PDZ domains. We find that 69 of the remaining 254 unmapped worm and human PDZ domains have greater than 70% binding-site identity to mapped domains, and thus are predicted to have near identical binding profiles (Figure 8 and Table S4). An analogous analysis using full-length PDZ domain sequences reveals that domains with greater than 50% overall identity also exhibit highly similar specificity profiles (Figure S2). Thus, by combining experimentally mapped and predicted PDZ domain binding specificities, we roughly double the size of our PDZ domain specificity map and achieve 45% coverage of 336 predicted worm and human PDZ domains. As these novel rules require only in silico analysis of primary sequence and perform well across worm and human, it should be possible to predict the specificity of a given PDZ domain sequence in any organism.

### Endogenous PDZ Interactions

One major application of our PDZ domain specificity map is protein interaction prediction. As previously observed for numerous PDZ domains, phage display selects high-affinity peptide ligands through an iterative panning process, some of which are physiologically relevant [20,29,30,39,40]. These studies have also demonstrated a strong correlation between phage-derived PWM scores and affinities determined for synthetic peptides. However, the in vivo ligand interactions for any given PDZ domain depend on its intrinsic peptide specificity, the concentration and context of the protein in which it is located, and the range and concentration of accessible ligands. Also, some ligands may interact with suboptimal affinities to regulate specific biological processes. Thus, endogenous C termini closely matching our mapped specificities are likely to bind the given PDZ domain in vitro, but determination of in vivo binding requires additional experimental support.

To significantly reduce the human PDZ interactome search space and prioritize interactions for future experimental testing, we detected the best matched C-terminal sequences in the human proteome for individual domains using a PWM-based scoring algorithm and a score threshold that stringently allows only the top few hits (Table S5). The network of potential human PDZ domain mediated protein interactions obtained in this manner contains 322 interactions between our 54 experimentally mapped PDZ domains and 228 human proteins. These high-scoring ligands are significantly enriched in known PDZ interactors (27 interactions are known, *p* = 8.6 × 10^−18^) (Table S5) and in gene function annotation consistent with known PDZ ligand-associated functions (Figure S3) [2–4]. Thus, our prioritized list is likely enriched in novel bona fide human PDZ protein interactions.

Our prioritization approach is useful because potential ligands can be considered for experimental follow-up in order of similarity to the phage-mapped specificity profile. For instance, when studying a particular protein of interest, it may be useful to expand the list of potential ligands to include additional lower-scoring ligands that may nonetheless be physiologically relevant. To illustrate the utility of this approach, we focused on DLG1, one of the first and best characterized PDZ-containing proteins (Table S6) [2,5,41], and extended the potential ligand list for the three DLG1 PDZ domains by choosing a less stringent score threshold. Our predicted interactions capture eight of the 11 known ligands for DLG1 (Table S7) and identify many additional potential ligands with scores comparable to those of the known ligands. The list includes many known ligands for the closely related DLG homologs (DLG2, −3, and −4) (Table S6), and is enriched in gene function annotations consistent with known functions of the DLG homologs (Figure S4), which are involved in establishing and maintaining cell polarity, and interact with ion channels, guanyl-nucleotide exchange factors, and other signal transduction proteins [2,5,41].

### Pathogenic PDZ Interactions

Pathogenic viruses and bacteria use short linear peptide motifs that target PDZ domains and other peptide-binding modules to perturb host signaling networks [8,10–13]. To study the extent of this pathogenic subversion of host cellular processes, we computationally identified 89 viral proteins with C termini matching mapped PDZ domain specificities better than the potential endogenous interactors defined above. These cover all PDZ domain specificity classes (Table S5). Our results suggest that many viruses specifically target distinct PDZ domain classes with high-affinity ligands that compete with endogenous interactors and interfere with normal physiology.

**Figure 9:**
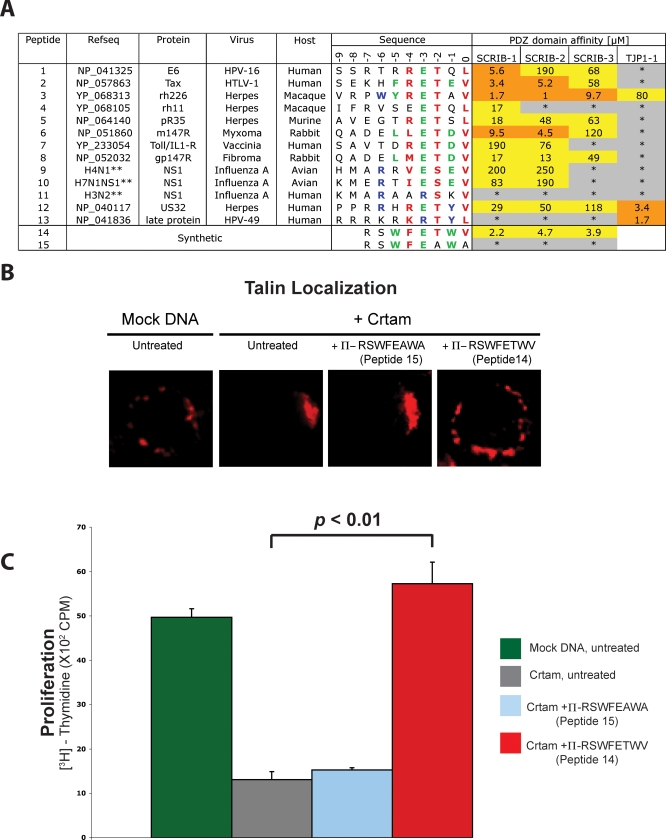
Viral Proteins Interfere with Host Cellular Function by Targeting the PDZ Domains of SCRIB (A) Many viral proteins bind SCRIB PDZ domains. Affinities were determined as IC_50_ values for peptides representing viral C termini binding to SCRIB PDZ domains and the first PDZ domain of ZO-1 (TJP1–1) [20]. Ligand sequence positions that match the specificity profiles for SCRIB, TJP1–1, or both, are colored green, blue, or red, respectively. Orange and yellow indicate high-affinity (IC_50_ < 10 μM) or moderate-affinity (IC_50_ > 10 μM) interactions, respectively. Asterisks (*) indicate no detectable interaction (IC_50_ > 500 μM). Double asterisks (**) indicate influenza A strain designations [13], rather than RefSeq accession numbers. (B) Loss of the late phase of T cell polarization induced by our designed synthetic peptide that targets SCRIB PDZ domains 1, 2, and 3. The receptor Crtam interacts with the PDZ domains of SCRIB to control cell growth and maintain polarity of T cells [40]. These effects are reversed by the addition of our designed peptide (Π-RSWFETWV, peptide 14) that binds with high affinity to the SCRIB PDZ domains, but not by a designed nonbinding peptide with mutations at the 0 and −2 positions (Π–RSWFEAWA, peptide 15). The symbol Π denotes the internalization sequence from the Antennapedia protein (RQIKIWFQNRRMKWKK), which has been shown to be internalized into cells [56]. Naive *Crtam^−/−^* CD4 T cells were electroporated with plasmid DNA expressing Crtam or a mock DNA control. Cells were treated with synthetic peptides (1.0 μM) and stained for Talin, a marker for the leading edge of polarized T cells [40]. (C) Our designed SCRIB PDZ-binding peptide (peptide 14) triggers T cell proliferation. Cells were treated with plasmid DNA and peptides, as described in (B), and cellular proliferation was measured by the incorporation of [^3^H]-thymidine. Data are representative of three independent experiments. Error bars indicate the standard deviation (SD). The *p*-value was determined by statistical analysis performed with a control using the Dunnett method.

To further explore viral targeting of PDZ domain proteins, we focused on SCRIB, a protein known to be targeted by human papilloma virus (HPV) [10]. SCRIB contains multiple PDZ domains and is involved in establishing and maintaining membrane polarity in epithelia, neurons, and T cells [4–6,40]. We identified numerous potential SCRIB viral ligands in our initial network and in an additional network derived from a recent database of avian influenza genomes [13]. We used a less stringent score cutoff because we desired a more focused and sensitive search designed to be experimentally validated. We measured in vitro affinities of ten potential SCRIB viral ligands using synthesized peptides (peptides 1–10, Figure 9A). Each peptide interacts with at least one SCRIB PDZ domain, but not, in general, with the first PDZ domain of ZO-1 (TJP1–1), which has an overlapping, but different, specificity [20]. As a further test, we found that a Herpes virus ligand (peptide 12) matching the specificity profiles of SCRIB and ZO-1 PDZ domains bound to both, while another ligand (peptide 13) matching only the ZO-1 PDZ domain specificity profile interacted only with this domain. These experiments show that our specificity map is useful to guide experiments and that viral proteins contain C-terminal motifs that are capable of specifically targeting distinct sets of PDZ domains.

Some of our predicted viral ligands are known, whereas many are novel. The HPV E6 protein (peptide 1) is known to disrupt SCRIB function and causes loss of epithelial cell polarization and concomitant hyperproliferation [10], and the PDZ-binding motif is only present in high-risk, oncogenic HPV strains. PDZ-binding motifs are also present in the Tax proteins of human T-lymphotrophic (HTLV) type 1 viruses (HTLV-1) (peptide 2) that cause adult T-cell leukemia/lymphoma (ATLL), but are absent from HTLV type 2 viruses that do not cause lymphoproliferative disorders [12]. The HTLV-1 Tax protein has been shown to interact with other PDZ domains [12], but we now show previously unreported interactions with SCRIB. In another example, the NS1 proteins of influenza A from avian and highly pathogenic human strains typically contain negatively charged residues at position −3 and can interact with many PDZ domains, whereas those from less pathogenic human strains typically contain positively charged residues at this position and show low reactivity with PDZ domains [13]. This is consistent with our specificity map (Figure 2) and with our binding data showing that the C termini of NS1 proteins from two avian influenza strains (peptides 9 and 10) interact with the SCRIB PDZ domains, but one from a low-pathogenicity human strain (peptide 11) does not. These confirmations, and new viral–SCRIB interactions we find involving herpes, vaccinia, myxoma, and fibroma viruses (peptides 3–8), suggest that many pathogenic viruses utilize a common mechanism to advantageously target SCRIB-mediated complexes involved in cell polarity and growth control.

To further explore this hypothesis, we investigated whether the PDZ-binding motif alone is sufficient to induce effects associated with pathogenic viral infections. Based on our PDZ specificity map, we designed a synthetic peptide that binds to the SCRIB PDZ domains with high affinity (peptide 14). We recently described an interaction between the C terminus (ESIV_COOH_) of the cell-surface receptor Crtam and SCRIB in T cells, which is critical to attenuate proliferation and maintain a late phase of T cell polarity [40]. Our designed SCRIB PDZ-binding peptide abrogates these functions and causes loss of late-phase T cell polarization and reverses the hypoproliferative effects of Crtam. In contrast, a nonbinding control peptide (peptide 15) has no effect (Figure 9). These effects are remarkably similar to the hyperproliferative phenotypes previously observed to be induced by the Tax protein of HTLV-1 in lymphocytes [12] and the E6 protein of high-risk HPV in epithelial cells [10]. The induction of hyperproliferation is likely to be advantageous for viral replication in general, and thus, it appears that the ability to disrupt polarity by interference with SCRIB PDZ domain complexes is a crucial factor in conferring high pathogenicity to many viruses, including HPV, HTLV, and influenza A.

## Discussion

We have presented the first large-scale specificity map of a domain family across species, based on approximately 3,100 peptide ligands, covering approximately one half of the combined set of 336 predicted PDZ domains encoded by the human and C. elegans genomes. We find that the PDZ domain family is surprisingly complex and diverse, recognizing up to seven C-terminal ligand residues and forming at least 16 unique specificity classes across human and worm. Further interpretation of our map reveals that PDZ domains are versatile, capable of binding diverse ligand sequence motifs, and are robust under high mutational load. Highly mutagenized WW and SH3 domain sequences also retain the ability to recognize proline-rich sequences [42,43], suggesting that functional robustness under high mutational pressure may be a general feature of peptide-binding modules. Although we find that the PDZ domain family likely evolved by domain expansion rather than from evolution of radically new specificities, the robustness of the PDZ domain may be ideal for supporting rapid evolution of interaction networks through testing of many functional variants under evolutionary pressures that select for novel ligands.

For the first time to our knowledge, we find a predictive correlation between PDZ domain sequence and binding specificity. This correlation bridges a gap in our ability to predict protein interactions and signaling networks from a genome. Because the correlation holds across worm and human, it can likely be used for accurate in silico predictions of PDZ domain specificity in other species. The predictive correlation will likely improve by considering additional features of the domain and ligand, including additional physicochemical and structural properties, class-specific binding sites, and cooperative and indirect effects of residues across the entire domain [44].

One major application of the PDZ domain specificity map is the prediction of interaction networks that provide insights into functions of PDZ domains in the cell. We have described a preliminary use of this map to prioritize human PDZ-mediated protein interactions, which directly led to novel insights into normal [40] and pathogen biology. Ideally, computational predictions would be more robust and less dependent on experimental support. A major impediment to domain-based protein interaction prediction is assessing predicted interaction validity, due to a dearth of bona fide PDZ ligands in the literature. This is further complicated because many interactions are known only for full-length proteins, often containing multiple PDZ domains. Thus, there are not enough known domain–peptide interactions to robustly learn optimal prediction parameters and accurately measure sensitivity and specificity. For instance, there is only one known worm interaction for our mapped PDZ domains, involving the lin-7 PDZ-containing protein that interacts with the C terminus of let-23 (the worm homolog of the epidermal growth factor receptor) [45], but this single interaction does agree with our data. Notwithstanding this limitation, higher-scoring potential interactors are more likely to be relevant, as supported by comparison to known PDZ domain interactions, analysis of gene function annotation, and comparison to well-studied examples [10,20,29,40]. This is not surprising, as similar computational methods with the same goal were successful in previously published PDZ domain studies [18,20,40]. Our list of prioritized interactions is a useful resource for biologists interested in further studying potential interactions involving PDZ domains. Ideally, this will lead to discovery of additional PDZ interactions that can be used to improve performance of computational protein interaction prediction methods.

The PDZ specificity map is useful for a number of applications. Our analysis reveals numerous viral proteins that may interact with PDZ domains to hijack host cellular networks for pathogen benefit. Based on our data, we were able to design synthetic viral-like peptides that target a specific biological system in human T cells. Analogously, therapeutics could be designed to alter PDZ-based cell systems for medical benefit [9], following approaches similar to those used for the development of peptidomimetics targeting other peptide-binding modules [46]. Our specificity map will prove invaluable for guiding peptidomimetic design, as it offers starting points for numerous PDZ domain specificity classes, provides optimal PDZ-binding peptides useful for target validation in cellular assays, and helps identify related domains and potential in vivo interaction partners that must be considered for cross-reactivity. Further, the versatility and robustness of the PDZ domain make it ideal for use in engineering synthetic biological systems [47].

Correct use and interpretation of our specificity map requires understanding of its physiological relevance. The map was constructed using optimal binding ligands detected by phage display. In the cell, however, natural ligands often bind suboptimally to enable regulation in signaling systems, and may have noncanonical binding modes. Multiple cellular factors must be considered to determine physiologically relevant binding using our data, including affinity, concentration, localization, and competition between similar PDZ domains for the same ligands.

Nevertheless, our C-terminal PDZ ligand dataset defines the diverse specificities of the PDZ domain family that have enabled the evolution of complex cellular architecture and provides a strong foundation for further work investigating physiologically relevant interactions. Further, the experimental and computational methods we describe are readily applicable to dozens of families of peptide recognition domains, covering a significant fraction of cell signaling proteins in eukaryotic genomes. We anticipate that derivation of specificity maps for all peptide recognition domains will enable the accurate prediction of physiologically relevant wiring diagrams directly from sequenced genomes.

## Materials and Methods

### PDZ domain identification.

For cloning, the domain boundaries were defined as the union of all domains found by a combination of the National Center for Biotechnology Information (NCBI) BLAST [31], PFAM [32], and SMART [33] with an additional ten amino acids on each side, as described previously [28]. For computational analysis, PDZ domain sequences were defined using hmmpfam precomputed by Ensembl [48] and downloaded from Ensembl 36 (homo_sapiens_core_36_35i and caenorhabditis_elegans_core_36_140c) using the Ensembl Perl API. Other domain resources were used to manually supplement this list when PDZ domains of interest were not found in Ensembl. Percent sequence identity was determined using a multiple sequence alignment of all human and worm PDZ domain sequences generated using MUSCLE 3.6 [49] with default parameters. Sequence identity was calculated as: number of matched positions divided by (aligned sequence length minus gap positions).

### PDZ domain cloning and expression.

DNA fragments encoding PDZ domains of interest (Tables S1 and S2) were amplified from cDNA using the polymerase chain reaction and were cloned into vectors designed for the expression and purification of PDZ domains fused to glutathione *S*-transferase, as described [20,28]. All expression vectors were verified by DNA sequencing.

### Selection of PDZ domain ligands.

C-terminal phage-displayed peptide libraries (>10^10^ unique members) were used to isolate ligands for PDZ domains using a series of iterative panning steps, as described [28]. Specific binding clones were individually tested for positive interactions with cognate PDZ domains by phage ELISA. Specific binding clones derived from sibling phages (identical in DNA sequence) were only counted once. This resulted in the isolation of 3,100 unique peptides from over 10,000 sequenced peptides for 82 PDZ domains. Data for six domains (ERBB2IP-1, SCRIB-1, SCRIB-2, SCRIB-3, TJP1–1, and TJP2–2) were from a previous study [20]. For other natural PDZ domains, a random decapeptide library was used in binding selections performed at room temperature. For the analysis of ERBB2IP-1 point mutants, a random heptapeptide library was used at 4 °C. A final manual inspection of all sequences removed a small number (92) that did not conform to the canonical C-terminal binding mode or did not agree with the major specificity profile (these sequences are available in our online files at http://baderlab.org/Data/PDZ).

### Specificity potential.

For each PDZ domain, the set of peptide ligands was used to create a binding profile statistical model as a PWM. The *SP* for a given column (position) of a PWM was calculated as is done for the letter height in a sequence logo [34], except normalized to range from 0 to 1 instead of 0 to 4.32 (log 20). A *SP* value of one means the given PDZ domain is completely specific for a single amino acid at that position, and a value of zero means that there is no preferred amino acid at that position. As no domains exhibited specificity at position −9, the *SP* value for each position was corrected for bias in the peptide library by subtracting the specificity score at position −9 for the entire set of 3,066 unique PDZ domain ligands (peptides found to bind to the 82 natural PDZ domains in the specificity map).

### Specificity map construction.

A specificity map (Figure 2) was constructed by clustering all 82 natural human and worm PDZ domain specificity profiles. We used hierarchical agglomerative clustering with average linkage with a custom specificity profile (PWM) distance metric, defined below. Each set of binding peptides was aligned, anchored by the C terminus, and used to create a specificity profile statistical model as a position weight matrix (PWM). Since the peptide library was constructed using a 32-codon set defined by the NNK nucleotide ambiguity codes, it is expected that some amino acids occur more frequently than others. To correct for this bias, the PWM was normalized by dividing amino acid frequencies by their expected frequency in the NNK codon set, following established practice [36]. To consider physicochemical similarities among amino acids and enable a more biologically relevant PWM similarity calculation, PWMs were recalculated to use a reduced amino acid alphabet of five groups constructed as follows: STQN (polar), KRH (basic), DE (acidic), FLAMPWIVCY (hydrophobic), and G. Distance between PWM pairs a and b, *D*(*a*,*b*), was then calculated using the following distance metric:


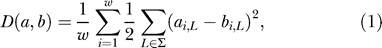


where *a* = PWM A; *b* = PWM B; *w* = number of columns in the PWM (i.e., ten–amino acids peptide length); ∑ = alphabet used in PWM = the number of rows in the PWM (i.e., five groups for the reduced alphabet defined above); and *L* = a letter from the reduced alphabet.

This distance metric is normalized such that zero represents perfectly similar PWMs and one represents perfectly dissimilar PWMs. Similarity is calculated as 1 − distance. The clustering results were visualized in standard fashion (as a tree with branch lengths corresponding to PWM pair distances). Leaf ordering for graphical tree display was optimized using the algorithm of Bar-Joseph et al. [50]. Clustered PWMs were graphically represented using sequence logos [34] and displayed as leaves on the cluster tree. Summary PWMs were constructed for all tree nodes as averages of all leaf node PWMs connected to that node and displayed on each tree node with a size proportional to their horizontal position in the tree. Software for creating this tree is available from http://baderlab.org/Software/LOLA. The tree was manually annotated by an expert (SSS) to define specificity classes.

### Erbin mutant heat map construction.

Each row of the heat map depicted in Figure 4 represents one of 91 Erbin mutants, and each column represents one of seven positions in the mutant specificity profile. To quantify the difference between mutant and wild-type profiles, both profiles were statistically modeled as PWMs and compared using the distance metric, *D*, described above, on a per PWM position basis (one position per heat map table cell). PWMs used 20 amino acids, instead of the reduced set described above, to provide a more fine-grained measure of PWM distance (which requires that the ∑ parameter in Equation 1 be the set of 20 amino acids). Resulting differences were then normalized across all 91 mutants per position (that is, over an entire column). The linear color gradient represents the difference of the mutant specificity profile compared to wild type, from minimum (blue) to maximum (red) distance. Significant differences are greater than one standard deviation away from the mean and highlighted in green. The map was then manually annotated with relevant sequence logos and structures.

### Prioritization of endogenous PDZ domain ligands.

The PWM representing the specificity profile for each of the 82 mapped PDZ domains was used to search for C-terminal matches in the RefSeq human proteome (∼33,700 proteins) and viral proteome (∼54,600 proteins) sets, downloaded May 21, 2007 [51]. SCRIB viral ligands were chosen in a more focused, but earlier, search of the RefSeq viral database from July 15, 2006, containing approximately 48,000 viral proteins and an additional set of influenza virus proteomes [13]. One pseudocount was added to each cell of the PWM to allow a low level of matching for amino acids that are not seen by phage display, but nevertheless, may be involved in a natural interaction. Matching potential ligands not having a hydrophobic C terminus were removed, since it is known from structural evidence that the PDZ domain is highly specific for hydrophobes at this position. A small number (∼10 ligands) were eliminated using this filter. PWM scores are calculated as the negative base 10 logarithm of the normalized probability of the PWM sequence match, such that low, positive scores represent better PWM matches. To enable comparisons across PWMs, scores were normalized to the range defined by the maximum and minimum possible scores that could be produced by the given PWM. Human proteome PWM score thresholds were calculated automatically for each PDZ domain by progressively testing increasing score thresholds and choosing a cutoff score when the number of new hits at a given threshold was higher than the cumulative number of hits of all previously tested thresholds (not including the last score tested). Viral PWM score cutoffs were defined to be better than the best human scoring match to ensure that only viral interactions with closer PWM matches than any human protein were predicted as viral PDZ ligands. Viral proteome matches in proteins that contained the string “phage” in their descriptions were removed, as these viruses likely target bacteria, not eukaryotic cells.

Additional ligands can be found by choosing a more liberal score threshold. We chose not to optimize the score threshold to maximize overlap of predicted interactions to a benchmark, since we could find no suitable benchmark. Available interactions in which the PDZ domain involved in the interaction was known were too few, and remaining interactions involved full-length proteins without domain-level resolution. We did not complete this conservative prioritization for worm due to the absence of almost any known interactions for worm PDZ domain containing proteins and poor Gene Ontology (GO) annotation coverage for predicted ligands.

The overlap statistic was computed based on all protein interactions involving our mapped PDZ domains in the UniHI database [52]. Approximately 8% (27) of prioritized interactions are known, which corresponds to a *p*-value of 8.6 × 10^−18^. The *p*-value was computed by calculating the overlap of 1,000 random shufflings of our prioritized interactions with the UniHI benchmark. Our randomly shuffled prioritized interactions overlapped approximately 6.7 interactions in the benchmark on average, with a standard deviation of approximately 3.4 and with a normal distribution. The normal distribution was used to calculate the *p*-value.

### Gene Ontology.

For predicted endogenous PDZ domain ligands, GO term enrichments were computed against all available GO annotation packaged with BiNGO on January 17, 2007, using the BiNGO Cytoscape plugin [53,54] with HUGO gene identifiers, the hypergeometric statistical test of significance, and Benjamini and Hochberg False Discovery Rate (FDR) correction with a significance level of 0.05.

### Software.

All computational analyses were performed using custom Java software built with BioJava 1.4 and JFreeChart. Free, open source Java software for visualizing and clustering specificity profiles is available from http://baderlab.org/Software/LOLA and for searching sequence databases using a specificity profile to find potential protein interactions via a Cytoscape plugin from http://baderlab.org/Software/BRAIN.

### Data.

All peptide sequences are available from http://baderlab.org/Data/PDZ and have been submitted to the DOMINO and PDZBase (accession codes cpe_3 to cpe_176) databases.

### Affinity assays.

Peptides were synthesized with acetylated C termini. The binding affinities of peptides for PDZ domains were determined as 50% inhibition concentration (IC_50_) values using competition ELISAs, as described [20]. The IC_50_ value was defined as the concentration of peptide that blocked 50% of PDZ domain binding to immobilized peptide.

### T cell assays.

Naïve *Crtam^−/−^* CD4 T cells were purified and activated with plate-bound anti-CD3 and anti-CD28, as described [40]. On day 4, T cells were electroporated with 4 μg of pIRES_GFP or pIRES_GFP/Crtam plasmid DNA by Amaxa Nucleofector (program X-01). Synthetic peptides (1.0 μM) were added into the cultures, and after 6 h, transfected cells were restimulated at 1 × 10^6^ cells/ml with plate-bound antibodies and were fixed 14 h later for Talin staining. After 42 h, [^3^H]-thymidine (1 μCi/well) was added, and the plates were harvested 8 h later.

## Supporting Information

Figure S1 Specificity Profiles for Point Mutants of ERBB2IP-1 Each column heading shows the wild-type sequence at each position, which is labeled according to a structure-based nomenclature shown in Figure 4 [36]. Each column shows the specificity profiles for the point mutants analyzed at that position, and the identity of each mutation is indicated to the left of each profile. The wild-type profile as observed at 4 °C is shown for comparison in the box at bottom left. The specificity profiles were derived from approximately 3,400 binding peptide sequences.
(3.85 MB PDF)

Figure S2 PDZ Domain Sequence Identity Accurately Predicts Binding Specificity The relationship between overall PDZ domain sequence identity and specificity profile similarity. Each point represents a pair of PDZ domains from our mapped set. Red circles represent pairs assigned to the same class, as defined in our specificity map, and blue squares represent all other pairs.
(267 KB PDF)

Figure S3 Gene Ontology Terms Associated with Endogenous Prioritized Human PDZ Ligands Overrepresented terms for the human proteins in Table S5 were calculated using the BiNGO plugin for Cytoscape and shown as circles [53,54]. Arrows connect less specific to more specific terms, as defined in GO. The area of a given node is proportional to the number of genes annotated in the corresponding GO category in our set of prioritized ligands. The node color scale is proportional to the *p-*value of the overrepresentation of the GO term in the set relative to the number of genes in the genome. White nodes are not significantly overrepresented, however they are included in order to illustrate the GO structure within the three different categories.
(A) GO biological process.
(B) GO molecular function.
(C) GO cellular localization.
(850 KB PDF)

Figure S4 Gene Ontology Terms Associated with Endogenous Predicted Ligands for the PDZ Domains of DLG1 Overrepresented GO biological process terms for the proteins in Table S6 were calculated using the BiNGO plugin for Cytoscape and shown as circles [53,54]. The analysis was performed as in Figure S3.
(270 KB PDF)

Table S1 Summary of Analyzed C. elegans PDZ Domains The domains are colored as follows: green, purified and peptide-phage selections were successful; blue, purified but peptide-phage selections were unsuccessful; grey, not cloned or could not be purified in a soluble form from Escherichia coli. The listed amino acid ranges indicate the length of the constructs used in the analysis and not necessarily the PDZ domain boundaries defined by computational domain identification.
(43 KB PDF)

Table S2 Summary of Analyzed Human PDZ Domains The domains are colored as follows: green, purified and peptide-phage selections were successful; blue, purified but peptide-phage selections were unsuccessful; grey, not cloned or could not be purified in a soluble form from E. coli. The listed amino acid ranges indicate the length of the constructs used in the analysis and not necessarily the PDZ domain boundaries defined by computational domain identification.
(47 KB PDF)

Table S3 *SP* Values for Human and C. elegans PDZ Domains Values were only determined for 72 domains that had ten or more selected peptides. C. elegans domains are highlighted in yellow, and values greater than or equal to 0.2 are highlighted in green.
(41 KB PDF)

Table S4 PDZ Domain Specificity Prediction Specificity of unmapped domains is predicted to be highly similar (>0.83 profile similarity) to the mapped domains with greater than 70% sequence identity in the binding site. The species of origin is shown to the right of each domain.
(9.07 MB PDF)

Table S5 Click here for additional data file. Prioritized Endogenous and Viral Ligands for Human PDZ Domains
PDZ domains are listed in alphabetical order and prioritized ligands are listed in ascending order by interaction score. Lower interaction scores are better. For each domain, only those viral ligands with better scores than the best endogenous ligand are shown. Viral or known endogenous ligands are highlighted in magenta or yellow, respectively.
(74 KB PDF)

Table S6 Prioritized Endogenous Ligands for the PDZ Domains of DLG1 PDZ domains are listed in numerical order, and for each, the 50 ligands with the best prediction score are listed in ascending order by interaction score. Lower interaction scores are better. Known ligands reported in PDZBase for DLG1 or the other DLG homologs (DLG2, −3, and −4) are highlighted in yellow or blue, respectively.
(41 KB PDF)

Table S7 Known Ligands for the PDZ Domains of DLG1 Known ligands reported in PDZBase are ordered in ascending order of interaction score for the highest scoring PDZ domain (Table S6). Lower interaction scores are better. *NA* denotes ligands that were not predicted by the scoring algorithm.
(24 KB PDF)

## Abbreviations

GO: Gene Ontology
HPV: human papilloma virus
HTLV: human T-lymphotrophic virus
IC50: 50% inhibition concentration
PDZ: post-synaptic density 95 (PSD-95)/Discs-large (Dlg)/zona occludens-1 (ZO-1)
PWM: position weight matrix
*SP*: specificity potential
