# Supplementary figures and images for: A Specificity Map for the PDZ Domain Family
Source: PLoS Biol. 2008 Sep 30;6(9):e239. doi: 10.1371/journal.pbio.0060239 (PMC2553845; doi:10.1371/journal.pbio.0060239)

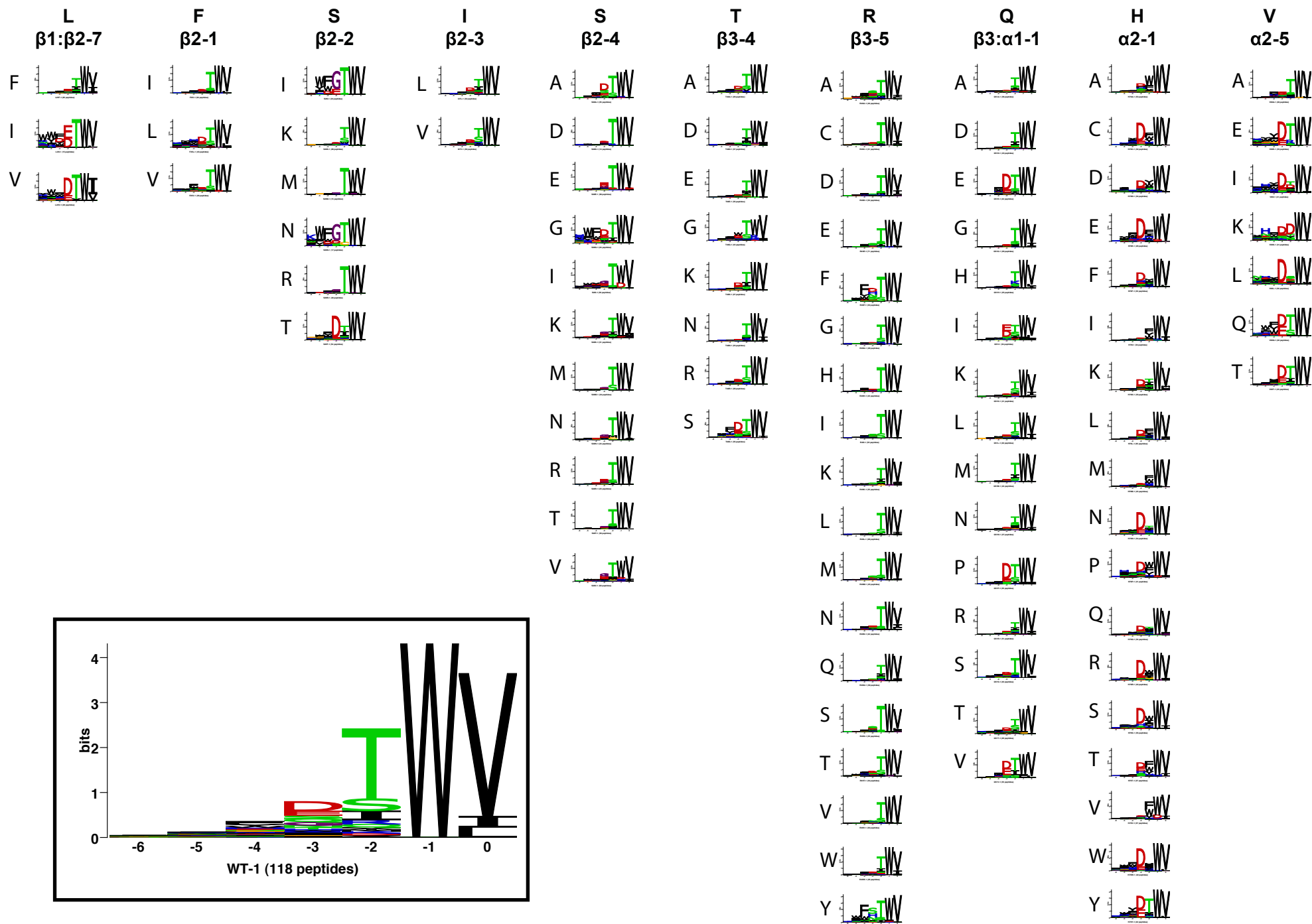

Supplement: Figure S1 — Each column heading shows the wild-type sequence at each position, which is labeled according to a structure-based nomenclature shown in Figure 4 [36]. Each column shows the specificity profiles for the point mutants analyzed at that position, and the identity of each mutation is indicated to the left of each profile. The wild-type profile as observed at 4 °C is shown for comparison in the box at bottom left. The specificity profiles were derived from approximately 3,400 binding peptide sequences. (3.85 MB PDF) [file pbio.0060239.sg001.pdf]

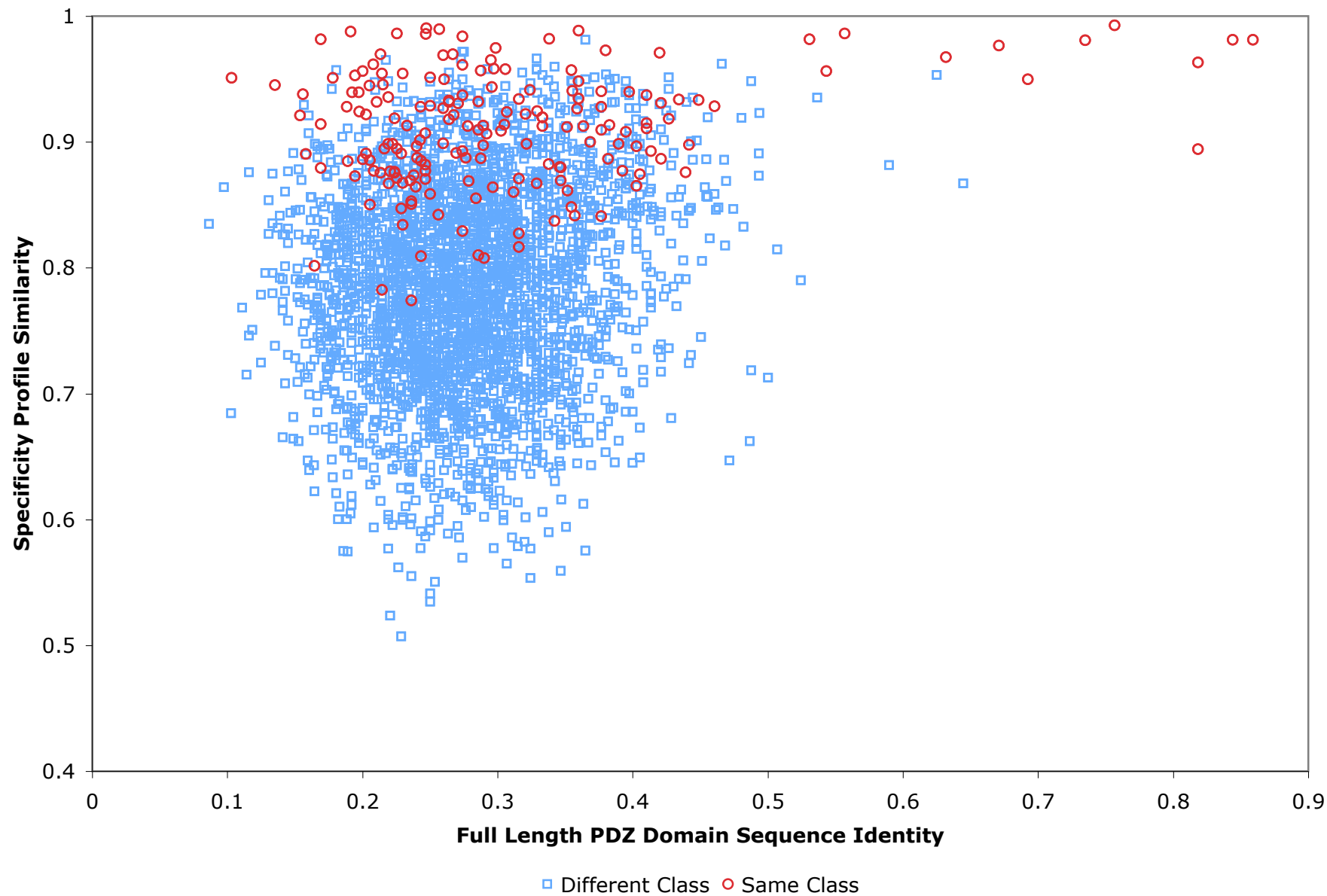

Supplement: Figure S2 — The relationship between overall PDZ domain sequence identity and specificity profile similarity. Each point represents a pair of PDZ domains from our mapped set. Red circles represent pairs assigned to the same class, as defined in our specificity map, and blue squares represent all other pairs. (267 KB PDF) [file pbio.0060239.sg002.pdf]

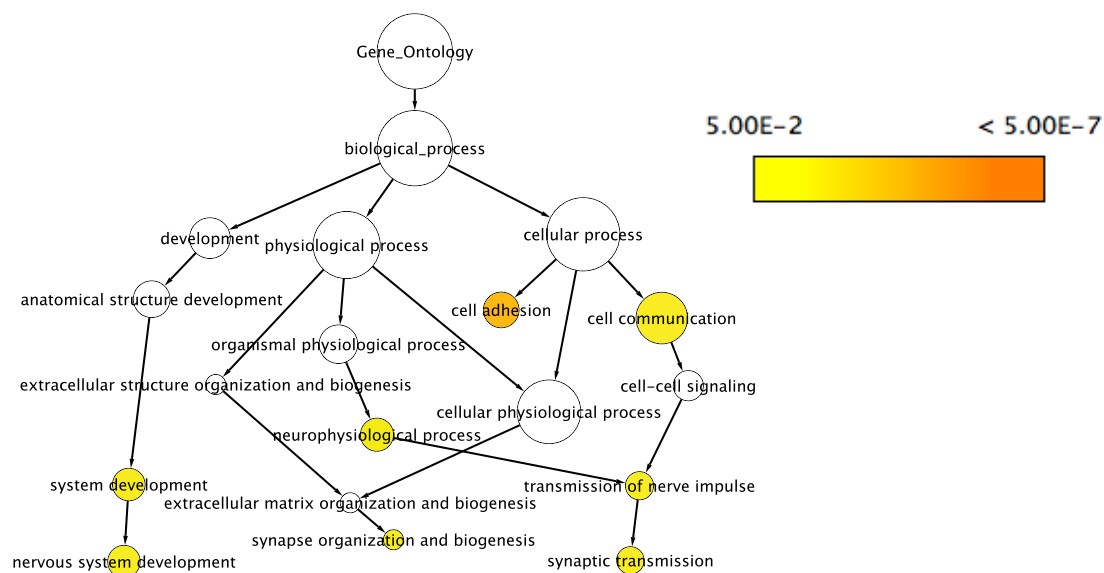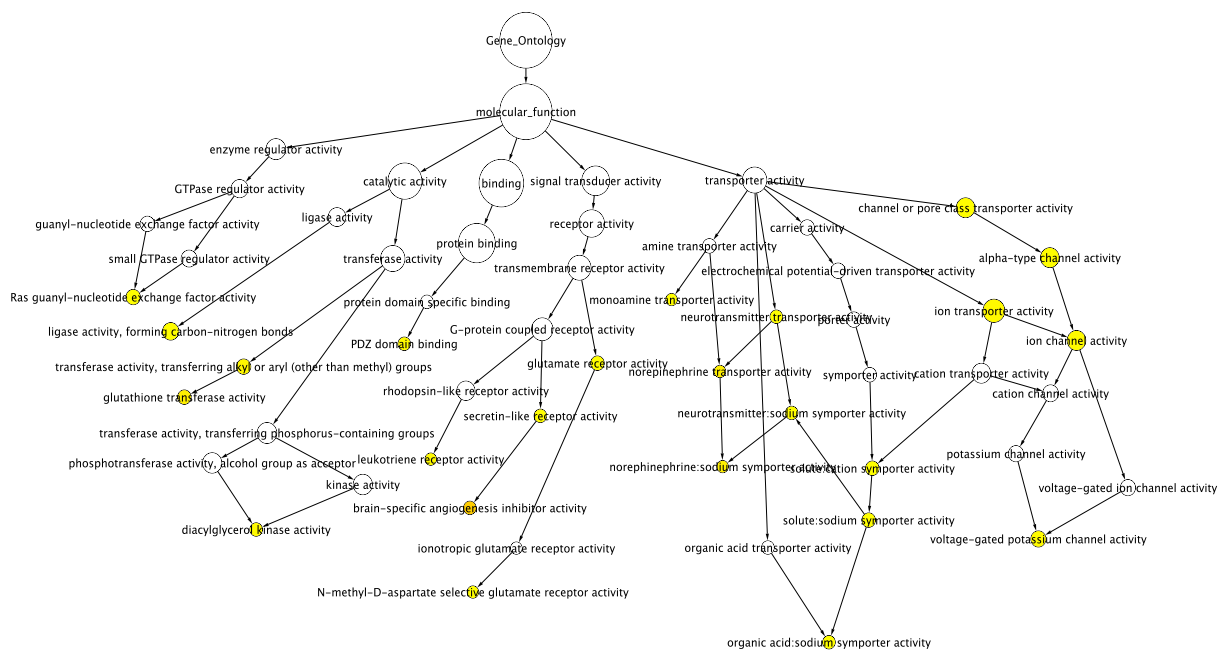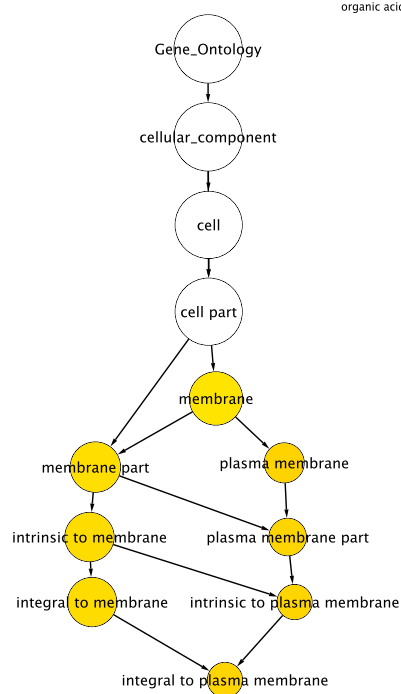

Supplement: Figure S3 — Overrepresented terms for the human proteins in Table S5 were calculated using the BiNGO plugin for Cytoscape and shown as circles [53,54]. Arrows connect less specific to more specific terms, as defined in GO. The area of a given node is proportional to the number of genes annotated in the corresponding GO category in our set of prioritized ligands. The node color scale is proportional to the p-value of the overrepresentation of the GO term in the set relative to the number of genes in the genome. White nodes are not significantly overrepresented, however they are included in order to illustrate the GO structure within the three different categories. (A) GO biological process. (B) GO molecular function. (C) GO cellular localization. (850 KB PDF) [file pbio.0060239.sg003.pdf]

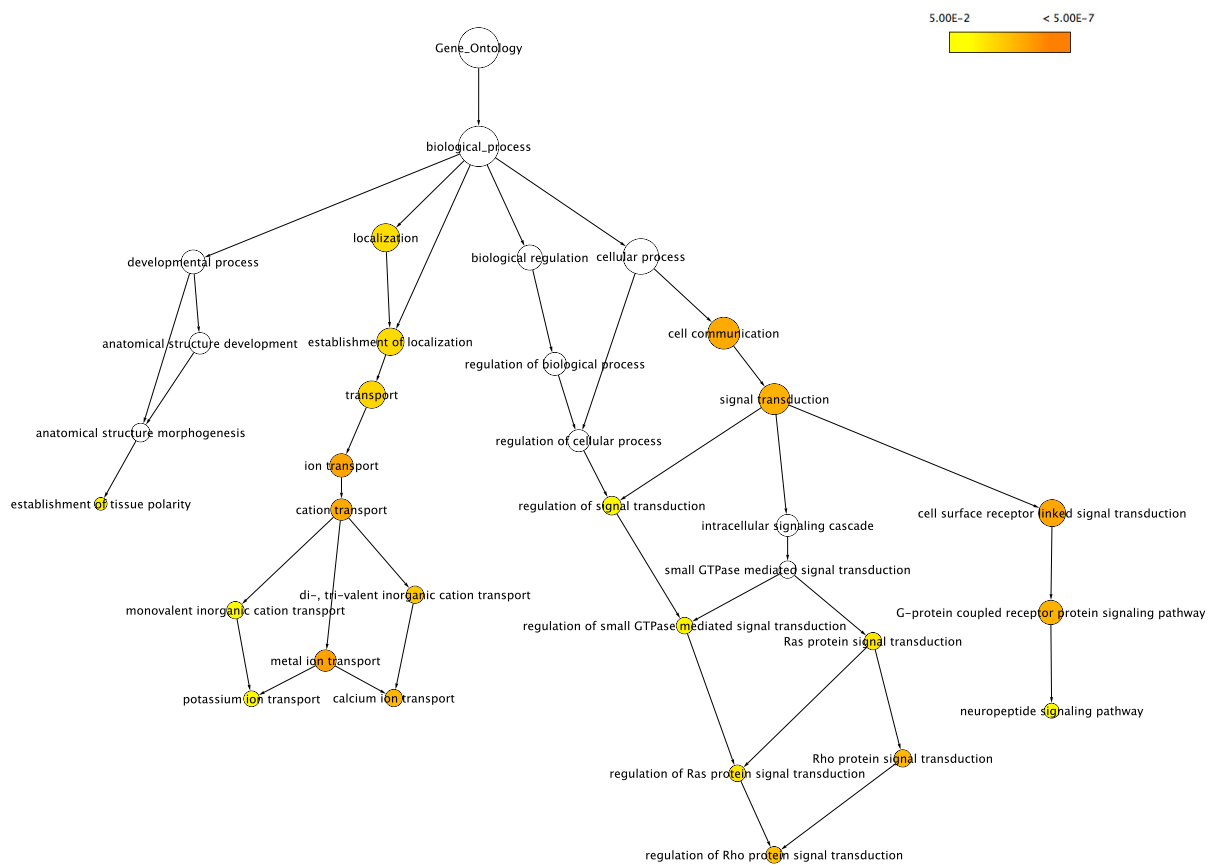

Supplement: Figure S4 — Overrepresented GO biological process terms for the proteins in Table S6 were calculated using the BiNGO plugin for Cytoscape and shown as circles [53,54]. The analysis was performed as in Figure S3. (270 KB PDF) [file pbio.0060239.sg004.pdf]
