# Supplementary material for: A Specificity Map for the PDZ Domain Family
Source: PLoS Biol. 2008 Sep 30;6(9):e239. doi: 10.1371/journal.pbio.0060239 (PMC2553845; doi:10.1371/journal.pbio.0060239)
Supplement: Table S1 — The domains are colored as follows: green, purified and peptide-phage selections were successful; blue, purified but peptide-phage selections were unsuccessful; grey, not cloned or could not be purified in a soluble form from Escherichia coli. The listed amino acid ranges indicate the length of the constructs used in the analysis and not necessarily the PDZ domain boundaries defined by computational domain identification. (43 KB PDF) [file pbio.0060239.st001.pdf]

Table S1. Summary of analyzed *C. elegans* PDZ domains.

The domains are colored as follows: green, purified and peptide-phage selections were successful; blue, purified but peptide-phage selections were unsuccessful; grey, not cloned or could not be purified in a soluble form from *E. coli*. The listed amino acid ranges indicate the length of the constructs used in the analysis and not necessarily the PDZ domain boundaries defined by computational domain identification.

	Wormpep ID	Gene (Common Name)	Transcript	PDZ Domain	Amino acid range	Purified	Peptides
1	WP:CE08042	C09G1.4	C09G1.4	C09G1.4_PDZ1	447-546	YES	YES
2	WP:CE37706	C11D9.1	C11D9.1	C11D9.1_PDZ1	107-205	YES	YES
3	WP:CE05307	C25G4.6	C25G4.6	C25G4.6_PDZ2	101-201	YES	YES
4	WP:CE01508	C33B4.3	C33B4.3	C33B4.3_PDZ1	425-539	YES	YES
5	WP:CE20599	C53B4.4a	C53B4.4a	C53B4.4a_PDZ1	491-597	YES	YES
6	WP:CE36524	dlg-1	C25F6.2a	dlg-1_PDZ3	497-601	YES	YES
7	WP:CE31680	dsh-1	C34F11.9a	dsh-1_PDZ1	291-401	YES	YES
8	WP:CE09647	F25H2.2	F25H2.2	F25H2.2_PDZ1	36 -152	YES	YES
9	WP:CE39293	frm-5	Y38C1AB.8	frm-5_PDZ3	1338-1434	YES	YES
10	WP:CE31024	frm-8	H09G03.2a	frm-8_PDZ1	84-182	YES	YES
11	WP:CE37387	K01A6.2	K01A6.2	K01A6.2_PDZ1	449-559	YES	YES
12	WP:CE37387	K01A6.2	K01A6.2	K01A6.2_PDZ3	745-851	YES	YES
13	WP:CE37387	K01A6.2	K01A6.2	K01A6.2_PDZ4	849-953	YES	YES
14	WP:CE37387	K01A6.2	K01A6.2	K01A6.2_PDZ5	995-1092	YES	YES
15	WP:CE20493	kin-4	C10C6.1	kin-4_PDZ1	1159-1268	YES	YES
16	WP:CE27131	lin-2	F17E5.1a	lin-2_PDZ1	533-637	YES	YES
17	WP:CE28370	lin-7	Y54G11A.10	lin-7_PDZ1	188-291	YES	YES
18	WP:CE35841	mpz-1	C52A11.4a	mpz-1_PDZ10	2071-2162	YES	YES
19	WP:CE35841	mpz-1	C52A11.4a	mpz-1_PDZ6	1203-1322	YES	YES
20	WP:CE35841	mpz-1	C52A11.4a	mpz-1_PDZ8	1718-1824	YES	YES
21	WP:CE35841	mpz-1	C52A11.4a	mpz-1_PDZ9	1818-1908	YES	YES
22	WP:CE08684	nab-1	C43E11.6a	nab-1_PDZ1	250-360	YES	YES
23	WP:CE28449	par-3	F54E7.3a	par-3_PDZ2	503-609	YES	YES
24	WP:CE28449	par-3	F54E7.3a	par-3_PDZ3	647-767	YES	YES
25	WP:CE13894	T21G5.4	T21G5.4	T21G5.4_PDZ1	1-91	YES	YES
26	WP:CE30169	unc-10	T10A3.1a	unc-10_PDZ1	633-745	YES	YES
27	WP:CE26729	W03F11.6a	W03F11.6a	W03F11.6a_PDZ1	958-1065	YES	YES
28	WP:CE29125	Y55B1BR.4	Y55B1BR.4	Y55B1BR.4_PDZ1	118-220	YES	YES
29	WP:CE03650	alp-1	T11B7.4a	alp-1_PDZ1	1-94	YES	NO
30	WP:CE06729	C01B7.5	C01B7.5	C01B7.5_PDZ1	22-120	YES	NO
31	WP:CE30847	C01F6.6a	C01F6.6a	C01F6.6a_PDZ2	131-235	YES	NO
32	WP:CE01529	C52A11.3	C52A11.3	C52A11.3_PDZ1	74 - 164	YES	NO
33	WP:CE36524	dlg-1	C25F6.2a	dlg-1_PDZ1	189-299	YES	NO
34	WP:CE36524	dlg-1	C25F6.2a	dlg-1_PDZ2	348-458	YES	NO
35	WP:CE27097	dsh-2	C27A2.6	dsh-2_PDZ1	349-458	YES	NO
36	WP:CE29779	F27D9.8a	F27D9.8a	F27D9.8a_PDZ1	53 - 160	YES	NO
37	WP:CE29296	F28F5.3a	F28F5.3a	F28F5.3a_PDZ1	316-418	YES	NO
38	WP:CE05865	F44D12.1	F44D12.1	F44D12.1_PDZ1	174-278	YES	NO
39	WP:CE05865	F44D12.1	F44D12.1	F44D12.1_PDZ2	265-357	YES	NO
40	WP:CE05865	F44D12.1	F44D12.1	F44D12.1_PDZ3	614-750	YES	NO
41	WP:CE24980	F45E4.3a	F45E4.3a	F45E4.3a_PDZ1	872-980	YES	NO
42	WP:CE39293	frm-5	Y38C1AB.8	frm-5_PDZ2	1238-1340	YES	NO
43	WP:CE37387	K01A6.2	K01A6.2	K01A6.2_PDZ2	563-663	YES	NO
44	WP:CE29778	let-413	F26D11.11a	let-413_PDZ1	562-674	YES	NO
45	WP:CE15610	lin-10	C09H6.2a	lin-10_PDZ1	791-898	YES	NO
46	WP:CE15610	lin-10	C09H6.2a	lin-10_PDZ2	880-978	YES	NO
47	WP:CE35841	mpz-1	C52A11.4a	mpz-1_PDZ5	780-888	YES	NO
48	WP:CE35841	mpz-1	C52A11.4a	mpz-1_PDZ7	1557-1659	YES	NO
49	WP:CE28449	par-3	F54E7.3a	par-3_PDZ1	371-495	YES	NO
50	WP:CE28089	par-6	T26E3.3	par-6_PDZ1	145-259	YES	NO
51	WP:CE17578	ptp-1	C48D5.2a	ptp-1_PDZ1	604 - 716	YES	NO
52	WP:CE09809	stn-1	F30A10.8a	stn-1_PDZ1	32-138	YES	NO
53	WP:CE39518	T19B10.5	T19B10.5	T19B10.5_PDZ1	225-328	YES	NO
54	WP:CE39292	Y38C1AB.4	Y38C1AB.4	Y38C1AB.4_PDZ2	1197-1299	YES	NO
55	WP:CE39292	Y38C1AB.4	Y38C1AB.4	Y38C1AB.4_PDZ3	1297-1393	YES	NO
56	WP:CE14968	Y57G11C.22	Y57G11C.22	Y57G11C.22_PDZ1	5-111	YES	NO

	Wormpep ID	Gene (Common Name)	Transcript	PDZ Domain	Amino acid range	Purified	Peptides
57	WP:CE16747	ZK849.1	ZK849.1	ZK849.1_PDZ1	202-310	YES	NO
58	WP:CE05307	C25G4.6	C25G4.6	C25G4.6_PDZ1	a.a 1-91	NO	NO
59	WP:CE08888	C50D2.3	C50D2.3	C50D2.3_PDZ1	33 - 135	NO	NO
60	WP:CE24021	T27F2.2	T27F2.2	T27F2.2_PDZ1	637-736	NO	NO
61	WP:CE01196	C35D10.2	C35D10.2	C35D10.2_PDZ1	NOT CLONED	NO	NO
62	WP:CE01858	C45G9.7	C45G9.7	C45G9.7_PDZ1	NOT CLONED	NO	NO
63	WP:CE32182	F23B2.8	F23B2.8	F23B2.8_PDZ1	NOT CLONED	NO	NO
64	WP:CE39293	frm-5	Y38C1AB.8	frm-5_PDZ1	NOT CLONED	NO	NO
65	WP:CE02318	mig-5	T05C12.6a	mig-5_PDZ1	NOT CLONED	NO	NO
66	WP:CE35841	mpz-1	C52A11.4a	mpz-1_PDZ1	NOT CLONED	NO	NO
67	WP:CE28080	pxf-1	T14G10.2a	pxf-1_PDZ1	NOT CLONED	NO	NO
68	WP:CE23996	T21C9.1	T21C9.1	T21C9.1_PDZ1	NOT CLONED	NO	NO
69	WP:CE39292	Y38C1AB.4	Y38C1AB.4	Y38C1AB.4_PDZ1	NOT CLONED	NO	NO
70	WP:CE25391	Y52E8A.1	Y52E8A.1	Y52E8A.1_PDZ1	NOT CLONED	NO	NO
71	WP:CE06728	C01B7.4	C01B7.4	C01B7.4_PDZ1	225-325	Insoluble	NO
72	WP:CE30847	C01F6.6a	C01F6.6a	C01F6.6a_PDZ1	1-104	Insoluble	NO
73	WP:CE25810	C44B7.1	C44B7.1	C44B7.1_PDZ1	65-177	Insoluble	NO
74	WP:CE38855	cnk-1	R01H10.8	cnk-1_PDZ1	252-355	Insoluble	NO
75	WP:CE30514	F23C8.13	F23C8.13	F23C8.13_PDZ1	1-82	Insoluble	NO
76	WP:CE27147	F30F8.3	F30F8.3	F30F8.3_PDZ1	45-156	Insoluble	NO
77	WP:CE32187	F44D12.4	F44D12.4	F44D12.4_PDZ1	128-219	Insoluble	NO
78	WP:CE35841	mpz-1	C52A11.4a	mpz-1_PDZ2	290-399	Insoluble	NO
79	WP:CE35841	mpz-1	C52A11.4a	mpz-1_PDZ3	430-541	Insoluble	NO
80	WP:CE35841	mpz-1	C52A11.4a	mpz-1_PDZ4	594-704	Insoluble	NO
81	WP:CE33391	syd-1	F35D2.5a	syd-1_PDZ1	112-218	Insoluble	NO
82	WP:CE13894	T21G5.4	T21G5.4	T21G5.4_PDZ2	101-201	Insoluble	NO
