# Supplementary material for: A Specificity Map for the PDZ Domain Family
Source: PLoS Biol. 2008 Sep 30;6(9):e239. doi: 10.1371/journal.pbio.0060239 (PMC2553845; doi:10.1371/journal.pbio.0060239)
Supplement: Table S2 — The domains are colored as follows: green, purified and peptide-phage selections were successful; blue, purified but peptide-phage selections were unsuccessful; grey, not cloned or could not be purified in a soluble form from E. coli. The listed amino acid ranges indicate the length of the constructs used in the analysis and not necessarily the PDZ domain boundaries defined by computational domain identification. (47 KB PDF) [file pbio.0060239.st002.pdf]

Table S2. Summary of analyzed human PDZ domains.

The domains are colored as follows: green, purified and peptide-phage selections were successful; blue, purified but peptide-phage selections were unsuccessful; grey, not cloned or could not be purified in a soluble form from *E. coli*. The listed amino acid ranges indicate the length of the constructs used in the analysis and not necessarily the PDZ domain boundaries defined by computational domain identification.

	Gene Description	HUGO Name	PDZ domain	Accession No.	Amino acid range	Purified	Peptide
1	Densin-180 PDZ	LRRC7	LRRC7_PDZ1	*NP476483.1*	1424-1537	YES	YES
2	DLG1 PDZ1	DLG1	DLG1_PDZ1	*NM_004087*	208-318	YES	YES
3	DLG1 PDZ2	DLG1	DLG1_PDZ2	*NM_004087*	311-422	YES	YES
4	DLG1 PDZ3	DLG1	DLG1_PDZ3	*NM_004087*	445-555	YES	YES
5	dvl2 PDZ	DVL2	DVL2_PDZ1	*AF006012*	248-365	YES	YES
6	Erbin PDZ	ERBB2IP	ERBB2IP_PDZ1	*NP061165.1*	1217-1371	YES	YES
7	Htra3	HRA3_HUMAN	HRA3_PDZ1	*AAP42283*	354-453	YES	YES
8	human AF6	MLLT4	MLLT4_PDZ1	*NM_005936*	991-1077	YES	YES
9	human CASK PDZ	CASK	CASK_PDZ1	*NM_003688*	478-582	YES	YES
10	Human neuroendocrine-dlg PDZ2	DLG3	DLG3_PDZ2	*U49089*	218-329	YES	YES
11	Human neuroendocrine-dlg PDZ3	DLG3	DLG3_PDZ3	*U49089*	368-475	YES	YES
12	human NHERF2 PDZ2	SLC9A3R2	SLC9A3R2_PDZ2	*AF035771*	136-242	YES	YES
13	human P55	MPP6	MPP6_PDZ1	*NM_016447*	109-220	YES	YES
14	human Par3 PDZ3	PARD3	PARD3_PDZ3	*NM_019619*	533-608	YES	YES
15	human PDZK1 PDZ1	PDZK1	PDZK1_PDZ1	*NM_002614*	1-106	YES	YES
16	human PDZK1 PDZ2	PDZK1	PDZK1_PDZ2	*NM_002614*	123-233	YES	YES
17	Human Shank3 PDZ2	Shank3	Shank3_PDZ2	*BC062987*	601-700	YES	YES
18	human SNTA1 PDZ	SNTA1	SNTA1_PDZ1	*NM_003098*	77-186	YES	YES
19	human TIAM	TIAM1	TIAM1_PDZ1	*NM_003253*	817-933	YES	YES
20	Human TIAM2 PDZ	TIAM2	TIAM2_PDZ	*NM_012454*	240-362	YES	YES
21	Human transcription factor PDZ	PSCDBP	PSCDBP_PDZ1	*L06633*	23-140	YES	YES
22	human Trithorax PDZ	MLLT4	MLLT4_PDZ1	*NM_005936*	967-1094	YES	YES
23	INADL PDZ2	INADL	INADL_PDZ2	*NP_795352*	235-355	YES	YES
24	INADL PDZ3	INADL	INADL_PDZ3	*NP_795352*	355-468	YES	YES
25	INADL PDZ6	INADL	INADL_PDZ6	*NP_795352*	1052-1179	YES	YES
26	LIN7 PDZ	LIN7A	LIN7A_PDZ	*NM_004664*	93-203	YES	YES
27	MAGI1 PDZ2	BAIAP1	BAIAP1_PDZ2	*NP_004733.1*	460-569	YES	YES
28	MAGI1 PDZ4	BAIAP1	BAIAP1_PDZ4	*NP_004733.1*	830-938	YES	YES
29	MAGI3 PDZ2	NP_066016	MAGI3_PDZ2	NP_066016	582-693	YES	YES
30	MAGI3 PDZ3	NP_066016	MAGI3_PDZ3	NP_066016	749-834	YES	YES
31	Multiple PDZ protein PDZ1	MPDZ	MPDZ_PDZ1	*NM_003829*	137-223	YES	YES
35	Multiple PDZ protein PDZ2	MPDZ	MPDZ_PDZ2	*NM_003829*	257-336	YES	YES
36	Multiple PDZ protein PDZ3	MPDZ	MPDZ_PDZ3	*NM_003829*	377-462	YES	YES
37	Multiple PDZ protein PDZ4	MPDZ	MPDZ_PDZ4	*NM_003829*	555-633	YES	YES
38	Multiple PDZ protein PDZ5	MPDZ	MPDZ_PDZ5	*NM_003829*	700-786	YES	YES

	Gene Description	HUGO Name	PDZ domain	Accession No.	Amino acid range	Purified	Peptide
39	Multiple PDZ protein PDZ7	MPDZ	MPDZ_PDZ7	*NM_003829*	1151-1246	YES	YES
40	Multiple PDZ protein PDZ9	MPDZ	MPDZ_PDZ9	*NM_003829*	1483-1563	YES	YES
32	Multiple PDZ protein PDZ10	MPDZ	MPDZ_PDZ10	*NM_003829*	1629-1711	YES	YES
33	Multiple PDZ protein PDZ12	MPDZ	MPDZ_PDZ12	*NM_003829*	1834-1919	YES	YES
34	Multiple PDZ protein PDZ13	MPDZ	MPDZ_PDZ13	*NM_003829*	1959-2042	YES	YES
41	OM1 (Htra2)	PRSS25	PRSS25_PDZ1	*AF141305*	359-458	YES	YES
42	PTPN13PDZ2	PTPN13	PTPN13_PDZ2	*NM_006264*	1338-1451	YES	YES
43	PTPN13PDZ4	PTPN13	PTPN13_PDZ4	*NM_006264*	1754-1864	YES	YES
44	PTPN4	PTPN4	PTPN4_PDZ	*BC010674*	517-589	YES	YES
45	Scribble PDZ1	SCRIB	SCRIB_PDZ1	*NP_056171.1*	718-829	YES	YES
46	Scribble PDZ2	SCRIB	SCRIB_PDZ2	*NP_056171.1*	857-983	YES	YES
47	Scribble PDZ3	SCRIB	SCRIB_PDZ3	*NP_056171.1*	982-1100	YES	YES
48	SeProtease (Htra1)	PRSS11	PRSS11_PDZ1	*NP_002766*	380-480	YES	YES
49	ZO1 PDZ1	TJP1	TJP1_PDZ1	*NM_003257*	414-506	YES	YES
50	ZO1 PDZ3	TJP1	TJP1_PDZ3	*NM_003257*	819-901	YES	YES
51	dvl1 PDZ	DVL1	DVL1_PDZ1	*HSU46461*	231-336	YES	NO
52	dvl3 PDZ	DVL3	DVL3_PDZ1	*NM_004423*	232-347	YES	NO
53	Human antigen NY-CO-38 PDZ1	USH1C	USH1C_PDZ1	*AF039700*	73-181	YES	NO
54	human APBA1 PDZ1	APBA1	APBA1_PDZ1	*NM_001163*	634-749	YES	NO
55	Human Chapsyn-110 PDZ3	DLG2	DLG2_PDZ3	*U32376*	400-514	YES	NO
56	human Enigma	PDLIM7	PDLIM7_PDZ1	*NM_005451*	1-112	YES	NO
57	human GRIP PDZ2	GRIP1	GRIP1_PDZ2	*AJ133439*	196-306	YES	NO
58	human GRIP PDZ3	GRIP1	GRIP1_PDZ3	*AJ133439*	298-404	YES	NO
59	human IL16 PDZ1	IL16	IL16_PDZ1	*NM_004513*	409-514	YES	NO
60	human LIMKinase	LIMK1	LIMK1_PDZ1	*NM_002314*	144-270	YES	NO
61	human NHERF PDZ2	SLC9A3R1	SLC9A3R1_PDZ2	*AF036241*	153-235	YES	NO
62	human P73 PDZ1	USH1C	USH1C_PDZ1	*NM_005709*	73-178	YES	NO
63	human P73 PDZ2	USH1C	USH1C_PDZ2	*NM_005709*	203-302	YES	NO
64	human P73 PDZ3	USH1C	USH1C_PDZ3	*NM_005709*	441-452	YES	NO
65	human PDZK1 PDZ3	PDZK1	PDZK1_PDZ3	*NM_002614*	231-334	YES	NO
66	human PDZK1 PDZ4	PDZK1	PDZK1_PDZ4	*NM_002614*	359-474	YES	NO
67	Human PDZ-LIM protein Mystique PDZ	PDLIM2	PDLIM2_PDZ1	*AY007729*	1-112	YES	NO
68	human PSCDBP	PSCDBP	PSCDBP_PDZ1	*NM_004288*	55-176	YES	NO
69	Human PSD95 PDZ3	DLG4	DLG4_PDZ3	*U83192*	336-444	YES	NO
70	human RIL	PDLIM4	PDLIM4_PDZ1	*NM_003687*	1-110	YES	NO
71	Human Tax interaction protein 43 PDZ	SNTB1	SNTB1_PDZ1	*AF028828*	10-110	YES	NO

	Gene Description	HUGO Name	PDZ domain	Accession No.	Amino acid range	Purified	Peptide
72	human TAX interaction protein PDZ	PAR6A	PAR6A_PDZ1	*AF028827*	28-150	YES	NO
73	Human TKA-1 PDZ2	NHERF2	NHERF2_PDZ2	*Z50150*	129-241	YES	NO
74	Human X104 PDZ2	TJP2	TJP2_PDZ2	*L27476*	286-394	YES	NO
75	Human X104 PDZ3	TJP2	TJP2_PDZ3	*L27476*	487-606	YES	NO
76	Human X11L2 PDZ1	APBA3	APBA3_PDZ1	*AB021638*	380-488	YES	NO
77	INADL PDZ5	INADL	INADL_PDZ5	*NP_795352*	675-799	YES	NO
78	INADL PDZ7	INADL	INADL_PDZ7	*NP_795352*	1222-1331	YES	NO
79	MAGI1 PDZ1	BAIAP1	BAIAP1_PDZ1	*NP_004733.1*	1-116	YES	NO
80	MAGI1 PDZ3	BAIAP1	BAIAP1_PDZ3	*NP_004733.1*	630-733	YES	NO
81	MPP1	MPP1	MPP1_PDZ1	*M64925*	71-1052	YES	NO
82	Multiple PDZ protein PDZ6	MPDZ	MPDZ_PDZ6	*NM_003829*	1008-1091	YES	NO
83	Multiple PDZ protein PDZ8	MPDZ	MPDZ_PDZ8	*NM_003829*	1350-1432	YES	NO
84	PTPN13PDZ1	PTPN13	PTPN13_PDZ1	*NM_006264*	1063-1177	YES	NO
85	PTPN13PDZ3	PTPN13	PTPN13_PDZ3	*NM_006264*	1467-1580	YES	NO
86	PTPN13PDZ5	PTPN13	PTPN13_PDZ5	*NM_006264*	1854-1958	YES	NO
87	Scribble PDZ4	SCRIB	SCRIB_PDZ4	*NP_056171.1*	1099-1219	YES	NO
88	ZO1 PDZ2	TJP1	TJP1_PDZ2	*NM_003257*	584-660	YES	NO
89	INADL PDZ1	INADL	INADL_PDZ1	*NP_795352*	111-240	NO	NO
91	INADL PDZ4	INADL	INADL_PDZ4	*NP_795352*	542-655	NO	NO
92	INADL PDZ8	INADL	INADL_PDZ8	*NP_795352*	1445-1520	NO	NO
93	INADL PDZ9	INADL	INADL_PDZ9	*NP_795352*	1542-1615	NO	NO
90	INADL PDZ10	INADL	INADL_PDZ10	*NP_795352*	1685-1762	NO	NO
94	MAGI1 PDZ5	BAIAP1	BAIAP1_PDZ5	*NP_004733.1*	988-1105	NO	NO
95	MAGI1 PDZ6	BAIAP1	BAIAP1_PDZ6	*NP_004733.1*	1141-1243	NO	NO
96	Multiple PDZ protein PDZ11	MPDZ	MPDZ_PDZ11	*NM_003829*	1725-1806	NO	NO
