# Supplementary material for: A Specificity Map for the PDZ Domain Family
Source: PLoS Biol. 2008 Sep 30;6(9):e239. doi: 10.1371/journal.pbio.0060239 (PMC2553845; doi:10.1371/journal.pbio.0060239)
Supplement: Table S3 — Values were only determined for 72 domains that had ten or more selected peptides. C. elegans domains are highlighted in yellow, and values greater than or equal to 0.2 are highlighted in green. (41 KB PDF) [file pbio.0060239.st003.pdf]

Table S3. SP values for human and *C. elegans* PDZ domains.

Values were determined for 72 domains that had 10 or more selected peptides.
C. elegans domains are highlighted in yellow and values greater than or equal to 0.2 are highlighted in green.

PDZ domain	Subsite *SP*										Total *SP*	Peptides
	-9	-8	-7	-6	-5	-4	-3	-2	-1	0		
MPDZ-4	0.00	0.00	0.00	0.44	0.23	0.88	0.88	0.98	0.88	0.98	5.3	14
INADL-3	0.00	0.00	0.00	0.32	0.25	0.98	0.61	0.74	0.98	0.76	4.6	15
F54E7.3a-3	0.00	0.40	0.12	0.73	0.29	0.56	0.59	0.98	0.14	0.78	4.6	13
TIAM1-1	0.00	0.00	0.00	0.13	0.00	0.92	0.69	0.98	0.92	0.92	4.6	30
CASK-1	0.00	0.00	0.00	0.00	0.93	0.02	0.71	0.98	0.89	0.93	4.5	38
PDLIM4-1	0.00	0.00	0.00	0.00	0.00	0.98	0.91	0.70	0.94	0.92	4.4	50
T10A3.1a-1	0.00	0.52	0.00	0.00	0.00	0.92	0.39	0.81	0.94	0.78	4.4	58
MPDZ-10	0.00	0.00	0.00	0.00	0.41	0.83	0.37	0.88	0.79	0.98	4.2	38
SCRIB-1	0.00	0.00	0.06	0.00	0.55	0.30	0.98	0.98	0.42	0.88	4.2	65
C34F11.9a-1	0.00	0.00	0.00	0.00	0.23	0.51	0.67	0.88	0.88	0.93	4.1	33
SCRIB-2	0.00	0.00	0.00	0.00	0.31	0.31	0.71	0.98	0.85	0.93	4.1	37
DLG1-3	0.00	0.00	0.00	0.00	0.44	0.73	0.50	0.90	0.73	0.76	4.1	18
LRRC7-1	0.00	0.00	0.03	0.00	0.50	0.25	0.69	0.90	0.98	0.69	4.0	71
INADL-6	0.00	0.00	0.00	0.00	0.27	0.32	0.75	0.86	0.98	0.86	4.0	10
MAGI1-4	0.00	0.00	0.00	0.06	0.00	0.90	0.58	0.67	0.98	0.81	4.0	46
DLG4-3	0.00	0.00	0.00	0.00	0.47	0.62	0.56	0.98	0.67	0.70	4.0	29
K01A6.2-4	0.00	0.00	0.00	0.00	0.00	0.74	0.62	0.86	0.98	0.75	3.9	10
DLG2-3	0.00	0.00	0.00	0.00	0.55	0.37	0.51	0.90	0.72	0.85	3.9	18
PDZK1-2	0.00	0.00	0.00	0.00	0.00	0.68	0.73	0.86	0.62	0.98	3.9	10
MPDZ-5	0.00	0.00	0.00	0.00	0.35	0.37	0.90	0.53	0.79	0.90	3.8	20
C09G1.4-1	0.00	0.34	0.00	0.62	0.40	0.00	0.47	0.36	0.93	0.70	3.8	32
MAGI3-3	0.00	0.00	0.00	0.00	0.00	0.98	0.71	0.68	0.91	0.53	3.8	60
MPDZ-12	0.00	0.00	0.00	0.00	0.00	0.66	0.30	0.98	0.87	0.98	3.8	22
W03F11.6a-1	0.00	0.00	0.00	0.33	0.64	0.88	0.38	0.62	0.30	0.42	3.6	35
Y55B1BR.4-1	0.00	0.00	0.00	0.00	0.00	0.98	0.24	0.74	0.85	0.72	3.5	45
MAGI1-2	0.00	0.00	0.00	0.00	0.75	0.49	0.00	0.91	0.60	0.77	3.5	69
TJP1-3	0.00	0.00	0.00	0.00	0.89	0.09	0.71	0.00	0.98	0.80	3.5	39
C33B4.3-1	0.00	0.00	0.00	0.00	0.00	0.53	0.72	0.92	0.57	0.69	3.4	66
K01A6.2-3	0.00	0.00	0.00	0.00	0.00	0.23	0.42	0.90	0.87	0.98	3.4	36
ERBB2IP-1	0.00	0.00	0.00	0.00	0.00	0.17	0.75	0.69	0.91	0.87	3.4	87
SCRIB-3	0.00	0.00	0.00	0.00	0.00	0.27	0.82	0.93	0.38	0.98	3.4	78
C43E11.6a-1	0.00	0.00	0.00	0.00	0.09	0.76	0.52	0.84	0.28	0.87	3.4	140
DVL2-1	0.00	0.00	0.00	0.00	0.22	0.32	0.57	0.98	0.54	0.70	3.3	34
PDZK1-1	0.00	0.00	0.00	0.12	0.19	0.16	0.73	0.85	0.38	0.88	3.3	38
K01A6.2-1	0.00	0.00	0.00	0.00	0.11	0.98	0.26	0.51	0.46	0.98	3.3	24
TJP2-3	0.00	0.00	0.00	0.00	0.60	0.55	0.72	0.00	0.91	0.48	3.3	52
F54E7.3a-2	0.00	0.00	0.00	0.51	0.53	0.29	0.39	0.14	0.81	0.54	3.2	17
INADL-2	0.00	0.00	0.00	0.00	0.00	0.29	0.72	0.58	0.72	0.77	3.1	16
MPDZ-13	0.00	0.00	0.00	0.00	0.07	0.16	0.50	0.82	0.73	0.80	3.1	59
DLG1-1	0.00	0.00	0.00	0.00	0.00	0.00	0.55	0.98	0.52	0.98	3.0	14
TJP1-1	0.00	0.00	0.00	0.33	0.00	0.22	0.31	0.78	0.65	0.65	2.9	43
APBA3-1	0.00	0.00	0.00	0.00	0.00	0.77	0.20	0.43	0.55	0.98	2.9	21
MPDZ-9	0.00	0.00	0.00	0.00	0.00	0.00	0.98	0.47	0.98	0.50	2.9	44
C52A11.4-10	0.00	0.00	0.00	0.00	0.00	0.00	0.86	0.47	0.64	0.92	2.9	26
DLG3-2	0.00	0.00	0.00	0.00	0.00	0.32	0.35	0.84	0.34	0.98	2.8	31
SLC9A3R2-2	0.00	0.00	0.00	0.00	0.00	0.00	0.57	0.89	0.56	0.68	2.7	44
C25G4.6-2	0.00	0.00	0.00	0.00	0.00	0.68	0.32	0.34	0.45	0.87	2.7	23
K01A6.2-5	0.00	0.00	0.00	0.00	0.00	0.00	0.00	0.87	0.98	0.76	2.6	22
Y38C1AB.4-2	0.00	0.00	0.00	0.00	0.00	0.82	0.15	0.48	0.36	0.79	2.6	64
Y54G11A.10-1	0.00	0.00	0.00	0.00	0.00	0.30	0.37	0.64	0.41	0.86	2.6	10
MAGI3-2	0.00	0.00	0.00	0.00	0.00	0.00	0.00	0.90	0.77	0.90	2.6	18
H09G03.2a-1	0.00	0.00	0.00	0.00	0.00	0.00	0.19	0.49	0.90	0.94	2.5	43
MPDZ-7	0.00	0.00	0.00	0.00	0.00	0.00	0.00	0.98	0.87	0.66	2.5	31
MPP6-1	0.00	0.00	0.00	0.00	0.00	0.00	0.00	0.72	0.83	0.86	2.4	20
SHANK3-1	0.00	0.00	0.00	0.00	0.00	0.00	0.39	0.91	0.42	0.68	2.4	67
MPDZ-2	0.00	0.00	0.00	0.00	0.00	0.14	0.39	0.43	0.83	0.51	2.3	59
T21G5.4-1	0.00	0.00	0.00	0.00	0.00	0.55	0.03	0.36	0.35	0.98	2.3	19
PDLIM2-1	0.00	0.00	0.00	0.00	0.00	0.00	0.00	0.22	0.98	0.94	2.1	49
SNTA1-1	0.00	0.00	0.00	0.00	0.00	0.00	0.00	0.98	0.63	0.52	2.1	16
PTPN13-2	0.00	0.00	0.00	0.00	0.00	0.00	0.00	0.76	0.32	0.98	2.1	26
PSCDBP-1	0.00	0.00	0.00	0.00	0.00	0.02	0.07	0.21	0.95	0.72	2.0	87
DLG1-2	0.00	0.00	0.00	0.00	0.00	0.07	0.15	0.85	0.03	0.87	2.0	57
C52A11.4-6	0.00	0.00	0.00	0.00	0.00	0.00	0.00	0.58	0.59	0.73	1.9	45
HTRA1-1	0.00	0.00	0.00	0.00	0.00	0.38	0.40	0.52	0.00	0.59	1.9	16
C11D9.1-1	0.00	0.00	0.00	0.00	0.00	0.02	0.33	0.79	0.08	0.65	1.9	85
F25H2.2-1	0.00	0.00	0.00	0.00	0.00	0.00	0.00	0.81	0.43	0.62	1.9	76
PTPN13-4	0.00	0.00	0.00	0.00	0.00	0.00	0.00	0.44	0.60	0.70	1.8	23
HTRA3-1	0.00	0.00	0.00	0.00	0.00	0.00	0.00	0.00	0.98	0.60	1.6	71
MPDZ-1	0.00	0.00	0.00	0.00	0.00	0.00	0.00	0.24	0.88	0.37	1.5	79
HTRA2-1	0.00	0.00	0.00	0.00	0.32	0.00	0.20	0.29	0.34	0.23	1.4	35
MPDZ-3	0.00	0.00	0.00	0.00	0.00	0.00	0.01	0.57	0.02	0.56	1.2	39
MLLT4-1	0.00	0.00	0.00	0.00	0.00	0.00	0.00	0.25	0.00	0.43	0.68	123
