# Supplementary material for: A Specificity Map for the PDZ Domain Family
Source: PLoS Biol. 2008 Sep 30;6(9):e239. doi: 10.1371/journal.pbio.0060239 (PMC2553845; doi:10.1371/journal.pbio.0060239)
Supplement: Table S4 — Specificity of unmapped domains is predicted to be highly similar (>0.83 profile similarity) to the mapped domains with greater than 70% sequence identity in the binding site. The species of origin is shown to the right of each domain. (9.07 MB PDF) [file pbio.0060239.st004.pdf]

Table S4. PDZ domain specificity prediction.

Specificity of unmapped domains is predicted to be highly similar (>0.83 profile similarity) to the mapped domains with greater than 70% sequence identity in the binding site. The species of origin is shown to the right of each domain.

Phage-derived Profile	Mapped	Species	Unmapped	Species	% Sequence Identity
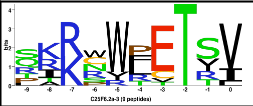	C25F6.2a-3	Worm	DLG3-3	Human	88
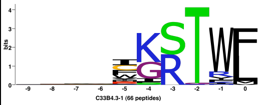	C33B4.3-1	Worm	ENST00000248851-1	Human	75
	C33B4.3-1	Worm	SHANK1-1	Human	75
	C33B4.3-1	Worm	SHANK2-1	Human	75
	C33B4.3-1	Worm	PDZK2-3	Human	73
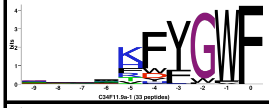	C34F11.9a-1	Worm	DVL1-1	Human	88
	C34F11.9a-1	Worm	DVL3-1	Human	88
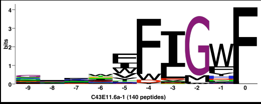	C43E11.6a-1	Worm	PPP1R9A-1	Human	81
	C43E11.6a-1	Worm	PPP1R9B-1	Human	81
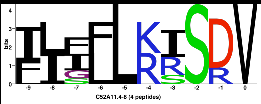	C52A11.4-8	Worm	INADL-8	Human	88
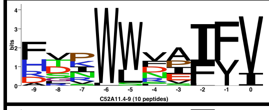	C52A11.4-9	Worm	INADL-9	Human	88
	C52A11.4-9	Worm	MPDZ-11	Human	88
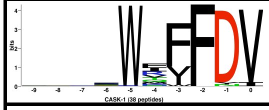	CASK-1	Human	MPP1-1	Human	75
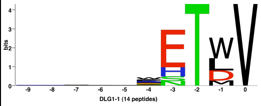	DLG1-1	Human	DLG2-1	Human	100
	DLG1-1	Human	DLG3-1	Human	100
	DLG1-1	Human	DLG4-1	Human	100
	DLG1-1	Human	C25F6.2a-2	Worm	94
	DLG1-1	Human	DLG2-2	Human	94
	DLG1-1	Human	DLG4-2	Human	94
	DLG1-1	Human	C25F6.2b.1-2	Worm	88
	DLG1-1	Human	C25F6.2a-1	Worm	81
	DLG1-1	Human	PDZK11-1	Human	75
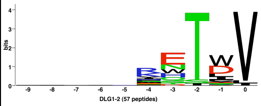	DLG1-2	Human	C25F6.2a-2	Worm	100
	DLG1-2	Human	DLG2-2	Human	100
	DLG1-2	Human	DLG4-2	Human	100
	DLG1-2	Human	C25F6.2b.1-2	Worm	94
	DLG1-2	Human	DLG2-1	Human	94
	DLG1-2	Human	DLG3-1	Human	94
	DLG1-2	Human	DLG4-1	Human	94
	DLG1-2	Human	C25F6.2a-1	Worm	88
	DLG1-2	Human	F30A10.8a-1	Worm	75
	DLG1-2	Human	LIN7B-1	Human	75
	DLG1-2	Human	LIN7C-1	Human	75

Phage-derived Profile	Mapped	Species	Unmapped	Species	% Sequence Identity
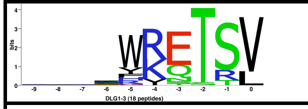	DLG1-3	Human	DLG3-3	Human	100
	DLG1-3	Human	LIN7B-1	Human	75
	DLG1-3	Human	LIN7C-1	Human	75
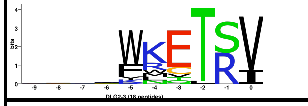	DLG2-3	Human	DLG3-3	Human	100
	DLG2-3	Human	LIN7B-1	Human	75
	DLG2-3	Human	LIN7C-1	Human	75
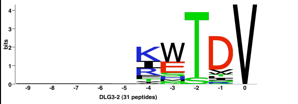	DLG3-2	Human	C25F6.2a-2	Worm	100
	DLG3-2	Human	DLG2-2	Human	100
	DLG3-2	Human	DLG4-2	Human	100
	DLG3-2	Human	C25F6.2b.1-2	Worm	94
	DLG3-2	Human	DLG2-1	Human	94
	DLG3-2	Human	DLG3-1	Human	94
	DLG3-2	Human	DLG4-1	Human	94
	DLG3-2	Human	C25F6.2a-1	Worm	88
	DLG3-2	Human	F30A10.8a-1	Worm	75
	DLG3-2	Human	LIN7B-1	Human	75
	DLG3-2	Human	LIN7C-1	Human	75
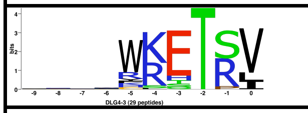	DLG4-3	Human	DLG3-3	Human	100
	DLG4-3	Human	LIN7B-1	Human	75
	DLG4-3	Human	LIN7C-1	Human	75
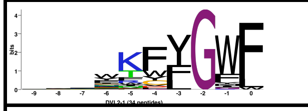	DVL2-1	Human	DVL1-1	Human	94
	DVL2-1	Human	DVL3-1	Human	94
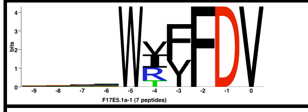	F17E5.1a-1	Worm	MPP1-1	Human	75
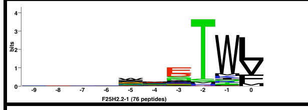	F25H2.2-1	Worm	C48D5.2a-1	Worm	75
	F25H2.2-1	Worm	SNX27-1	Human	75
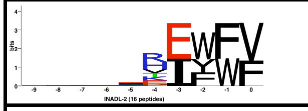	INADL-2	Human	C52A11.4-1	Worm	81
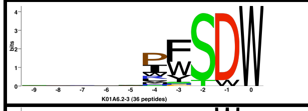	K01A6.2-3	Worm	MAGI1-3	Human	73
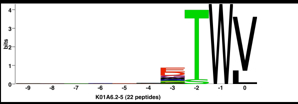	K01A6.2-5	Worm	AIP1_HUMAN-6	Human	88
	K01A6.2-5	Worm	MAGI1-5	Human	81
	K01A6.2-5	Worm	MAGI3-5	Human	81
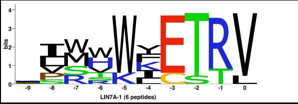	LIN7A-1	Human	LIN7B-1	Human	94
	LIN7A-1	Human	LIN7C-1	Human	94
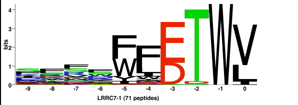	LRRC7-1	Human	C25F6.2a-1	Worm	75
	LRRC7-1	Human	C25F6.2a-2	Worm	75
	LRRC7-1	Human	DLG2-2	Human	75
	LRRC7-1	Human	DLG4-2	Human	75
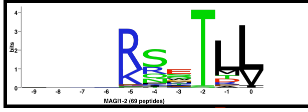	MAGI1-2	Human	AIP1_HUMAN-3	Human	73
	MAGI1-2	Human	K01A6.2-2	Worm	73

Phage-derived Profile	Mapped	Species	Unmapped	Species	% Sequence Identity
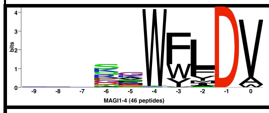	MAGI1-4	Human	NP_001028229.1-4	Human	100
	MAGI1-4	Human	AIP1_HUMAN-5	Human	94
	MAGI1-4	Human	MAGI3-4	Human	88
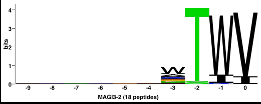	MAGI3-2	Human	AIP1_HUMAN-3	Human	75
	MAGI3-2	Human	K01A6.2-2	Worm	75
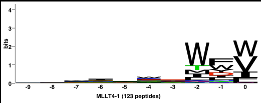	MLLT4-1	Human	NP_005927.1-1	Human	100
	MLLT4-1	Human	W03F11.6e-1	Worm	85
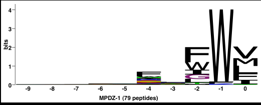	MPDZ-1	Human	INADL-1	Human	81
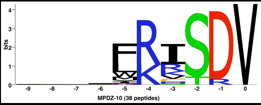	MPDZ-10	Human	INADL-8	Human	88
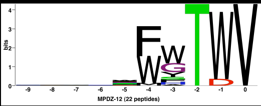	MPDZ-12	Human	INADL-10	Human	82
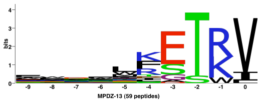	MPDZ-13	Human	LNX2-4	Human	81
	MPDZ-13	Human	NP_757366.1-1	Human	81
	MPDZ-13	Human	C25F6.2a-1	Worm	75
	MPDZ-13	Human	C25F6.2a-2	Worm	75
	MPDZ-13	Human	DLG2-2	Human	75
	MPDZ-13	Human	DLG4-2	Human	75
	MPDZ-13	Human	F30A10.8a-1	Worm	75
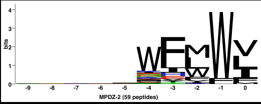	MPDZ-2	Human	C52A11.4-1	Worm	88
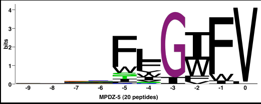	MPDZ-5	Human	INADL-5	Human	94
	MPDZ-5	Human	C52A11.4-4	Worm	88
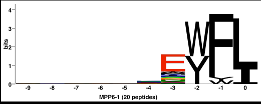	MPP6-1	Human	MPP2-1	Human	80
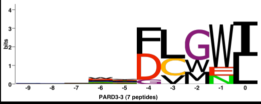	PARD3-3	Human	ALS2CR19-3	Human	88
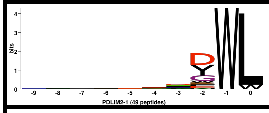	PDLIM2-1	Human	PDLIM3-1	Human	71
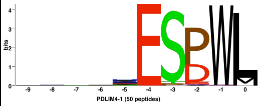	PDLIM4-1	Human	PDLIM1-1	Human	88
	PDLIM4-1	Human	LDB3-1	Human	82
	PDLIM4-1	Human	PDLIM5-1	Human	75
	PDLIM4-1	Human	PDLIM3-1	Human	71
	PDLIM4-1	Human	PDLIM7-1	Human	71

Phage-derived Profile	Mapped	Species	Unmapped	Species	% Sequence Identity
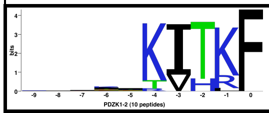	PDZK1-2	Human	ENST00000339068-1	Human	93
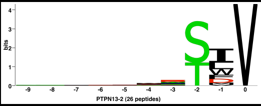	PTPN13-2	Human	NP_542415.1-2	Human	94
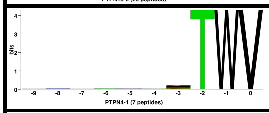	PTPN4-1	Human	C48D5.2a-1	Worm	94
	PTPN4-1	Human	PTPN3-1	Human	75
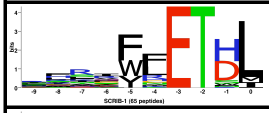	SCRIB-1	Human	SCRIB-4	Human	75
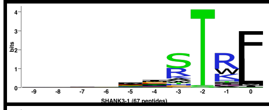	SHANK3-1	Human	ENST00000248851-1	Human	100
	SHANK3-1	Human	SHANK1-1	Human	94
	SHANK3-1	Human	SHANK2-1	Human	94
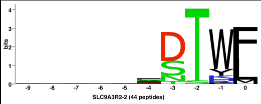	SLC9A3R2-2	Human	SLC9A3R1-2	Human	87
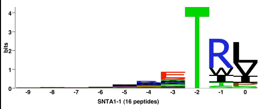	SNTA1-1	Human	SNTB1-1	Human	100
	SNTA1-1	Human	SNTB2-1	Human	100
	SNTA1-1	Human	F30A10.8a-1	Worm	94
	SNTA1-1	Human	F27D9.8a-1	Worm	88
	SNTA1-1	Human	SNTG2-1	Human	81
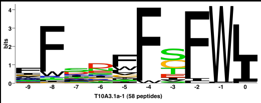	T10A3.1a-1	Worm	RIMS2-1	Human	80
	T10A3.1a-1	Worm	RIMS1_HUMAN-1	Human	73
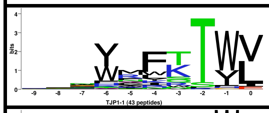	TJP1-1	Human	NP_783297.1-1	Human	100
	TJP1-1	Human	TJP2-1	Human	88
	TJP1-1	Human	TJP3-1	Human	75
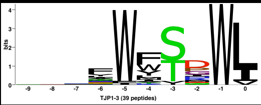	TJP1-3	Human	TJP3-3	Human	76
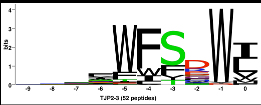	TJP2-3	Human	TJP3-3	Human	82
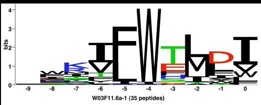	W03F11.6a-1	Worm	W03F11.6e-1	Worm	100
	W03F11.6a-1	Worm	NP_005927.1-1	Human	88
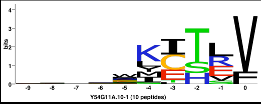	Y54G11A.10-1	Worm	LIN7B-1	Human	94
	Y54G11A.10-1	Worm	LIN7C-1	Human	94
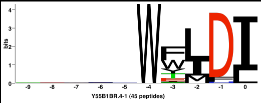	Y55B1BR.4-1	Worm	MPP4-1	Human	73
	Y55B1BR.4-1	Worm	MPP7-1	Human	73
