# Supplementary material for: A Specificity Map for the PDZ Domain Family
Source: PLoS Biol. 2008 Sep 30;6(9):e239. doi: 10.1371/journal.pbio.0060239 (PMC2553845; doi:10.1371/journal.pbio.0060239)
Supplement: Table S5 [file pbio.0060239.st005.pdf]

Table S5. Prioritized endogenous and viral ligands for human PDZ domains.

PDZ domains are listed in alphabetical order and prioritized ligands are listed in ascending order by interaction score. Lower interaction scores are better. For each domain, only those viral ligands with better scores than the best endogenous ligand are shown. Viral or known endogenous ligands are highlighted in magenta or yellow, respectively.

	PDZ domain	Prioritized ligand	Interaction score	RefSeq ID	C terminal motif	Origin
1	APBA3-1	NP_077713	4.40	NP_077713	VRYKYFNYMC	Viral
2	APBA3-1	NP_477779	4.68	NP_477779	SIFLFFDFFI	Viral
3	APBA3-1	NP_068345	4.75	NP_068345	KIENTFNFNF	Viral
4	APBA3-1	YP_164199	4.92	YP_164199	EPSSFFDPFF	Viral
5	APBA3-1	ARHGAP11A	4.97	NP_955389	PKKGTFTYYC	Human
6	APBA3-1	LOC728012	5.92	XP_001126358	KTFIWFKDFC	Human
7	APBA3-1	LOC731849	5.92	XP_001131032	KTFIWFKDFC	Human
8	CASK-1	NP_044656	5.73	NP_044656	QEIQRRLFDV	Viral
9	CASK-1	PARK2	5.92	NP_004553	VCMGDHWFDV	Human
10	DLG1-1	NP_613150	1.73	NP_613150	FVDVVRETLV	Viral
11	DLG1-1	NP_689241	1.73	NP_689241	FVNVVRETLV	Viral
12	DLG1-1	NP_052032	1.95	NP_052032	QADELMETDV	Viral
13	DLG1-1	YP_803243	2.07	YP_803243	NDLFFSETLV	Viral
14	DLG1-1	NP_042202	2.22	NP_042202	SVVTDRETDV	Viral
15	DLG1-1	NP_536584	2.22	NP_536584	SAVTDRETDV	Viral
16	DLG1-1	NP_570556	2.22	NP_570556	SAVTDRETDV	Viral
17	DLG1-1	NP_619966	2.22	NP_619966	SAVTDRETDV	Viral
18	DLG1-1	NP_671664	2.22	NP_671664	SAVTDRETDV	Viral
19	DLG1-1	YP_233054	2.22	YP_233054	SAVTDRETDV	Viral
20	DLG1-1	YP_717479	2.22	YP_717479	SAVTDRETDV	Viral
21	DLG1-1	PBK	2.25	NP_060962	HIVEALETDV	Human
22	DLG1-1	ARHGAP6	2.41	NP_038286	NPDALPETLV	Human
23	DLG1-1	NET1	2.45	NP_005854	SGGKRKETLV	Human
24	DLG1-1	KCNA4	2.51	NP_002224	SNAKAVETDV	Human
25	DLG1-1	ARHGEF16	2.64	NP_055263	MERLRVETDV	Human
26	DLG1-1	ABR	2.86	NP_068781	RNTLYFSTDV	Human
27	DLG1-1	LOC732425	2.86	XP_001133480	GQSILFSTDV	Human
28	DLG1-1	TMEM16J	3.00	NP_001012302	SIFSARSTDV	Human
29	DLG1-2	NP_613150	1.53	NP_613150	FVDVVRETLV	Viral
30	DLG1-2	NP_689241	1.53	NP_689241	FVNVVRETLV	Viral
31	DLG1-2	NP_049561	1.58	NP_049561	AEKHFRETEV	Viral
32	DLG1-2	NP_042202	1.59	NP_042202	SVVTDRETDV	Viral
33	DLG1-2	NP_536584	1.60	NP_536584	SAVTDRETDV	Viral
34	DLG1-2	NP_570556	1.60	NP_570556	SAVTDRETDV	Viral
35	DLG1-2	NP_619966	1.60	NP_619966	SAVTDRETDV	Viral
36	DLG1-2	NP_671664	1.60	NP_671664	SAVTDRETDV	Viral
37	DLG1-2	YP_233054	1.60	YP_233054	SAVTDRETDV	Viral
38	DLG1-2	YP_717479	1.60	YP_717479	SAVTDRETDV	Viral
39	DLG1-2	YP_309009	1.73	YP_309009	IKEKIINTIV	Viral
40	DLG1-2	NP_057863	1.76	NP_057863	SEKHFRETEV	Viral
41	DLG1-2	NP_057864	1.76	NP_057864	SEKHFRETEV	Viral
42	DLG1-2	LOC401498	1.97	NP_997723	QGRWDHETIV	Human
43	DLG1-2	TMEM16J	2.06	NP_001012302	SIFSARSTDV	Human
44	DLG1-2	NET1	2.21	NP_005854	SGGKRKETLV	Human
45	DLG1-2	KCNA4	2.33	NP_002224	SNAKAVETDV	Human
46	DLG1-2	PBK	2.41	NP_060962	HIVEALETDV	Human
47	DLG1-2	MARCH3	2.41	XP_001127871	VKRNSKETVV	Human
48	DLG1-2	CRIPT	2.46	NP_054890	DTKNYKQTSV	Human
49	DLG1-2	PLEKHA2	2.50	XP_945579	DDENIRTSDV	Human
50	DLG1-2	ADRA1D	2.55	NP_000669	DYSNLRETDI	Human

	PDZ domain	Prioritized ligand	Interaction score	RefSeq ID	C terminal motif	Origin
51	DLG1-2	KIF1B	2.59	NP_904325	NLKAGRETTV	Human
52	DLG1-2	ARHGEF16	2.64	NP_055263	MERLRVETDV	Human
53	DLG1-2	ERBB4	2.68	NP_001036064	PPYRHRNTVV	Human
54	DLG1-2	KCNA5	2.69	NP_002225	CLDTSRETDL	Human
55	DLG1-2	GRIN2A	2.76	NP_000824	KKMPSIESDV	Human
56	DLG1-2	DGKZ	2.79	NP_963290	IQREDQETAV	Human
57	DLG1-2	LOC285382	2.82	NP_001020437	EVIETIETTV	Human
58	DLG1-2	C1orf76	2.86	NP_775780	TNPRAISTDV	Human
59	DLG1-2	PLAC8L1	2.92	NP_001025040	AVPMTKDTLV	Human
60	DLG1-2	FRMPD4	2.94	NP_055543	RLPKIKETTV	Human
61	DLG1-2	LRRC3B	2.94	NP_443185	DEPDDISTVV	Human
62	DLG1-2	LOC732425	2.97	XP_001133480	GQSILFSTDV	Human
63	DLG1-2	ABR	2.99	NP_068781	RNTLYFSTDV	Human
64	DLG1-3	CRIPT	1.99	NP_054890	DTKNYKQTSV	Human
65	DLG1-3	CYSLTR2	3.43	NP_065110	SVWLRKETRV	Human
66	DLG1-3	KIF1B	3.50	NP_904325	NLKAGRETTV	Human
67	DLG1-3	GUCY1A2	4.43	NP_000846	GTMFLRETSL	Human
68	DLG1-3	TRPV3	4.56	NP_659505	EVEEFPETSV	Human
69	DLG1-3	FRMPD4	4.69	NP_055543	RLPKIKETTV	Human
70	DLG2-3	NP_477767	2.61	NP_477767	FKKFFWVTRV	Viral
71	DLG2-3	NP_477768	2.61	NP_477768	FKKFFWVTRV	Viral
72	DLG2-3	CYSLTR2	2.71	NP_065110	SVWLRKETRV	Human
73	DLG2-3	CRIPT	2.99	NP_054890	DTKNYKQTSV	Human
74	DLG3-2	NP_042202	2.04	NP_042202	SVVTDRETDV	Viral
75	DLG3-2	NP_536584	2.04	NP_536584	SAVTDRETDV	Viral
76	DLG3-2	NP_570556	2.04	NP_570556	SAVTDRETDV	Viral
77	DLG3-2	NP_619966	2.04	NP_619966	SAVTDRETDV	Viral
78	DLG3-2	NP_671664	2.04	NP_671664	SAVTDRETDV	Viral
79	DLG3-2	YP_233054	2.04	YP_233054	SAVTDRETDV	Viral
80	DLG3-2	YP_717479	2.04	YP_717479	SAVTDRETDV	Viral
81	DLG3-2	GRIN2A	2.24	NP_000824	KKMPSIESDV	Human
82	DLG3-2	TMEM16J	2.31	NP_001012302	SIFSARSTDV	Human
83	DLG3-2	MARCH3	2.43	XP_001127871	VKRNSKETVV	Human
84	DLG3-2	C1orf76	2.45	NP_775780	TNPRAISTDV	Human
85	DLG3-2	LRRC3B	2.46	NP_443185	DEPDDISTVV	Human
86	DLG3-2	LOC285382	2.48	NP_001020437	EVIETIETTV	Human
87	DLG3-2	KCNA4	2.60	NP_002224	SNAKAVETDV	Human
88	DLG3-2	ABR	2.62	NP_068781	RNTLYFSTDV	Human
89	DLG3-2	LOC732425	2.63	XP_001133480	GQSILFSTDV	Human
90	DLG3-2	ARHGEF16	2.72	NP_055263	MERLRVETDV	Human
91	DLG3-2	FRMPD4	2.73	NP_055543	RLPKIKETTV	Human
92	DLG3-2	LOC644165	2.82	XP_938794	RQSILFSTDV	Human
93	DLG3-2	LOC731634	2.82	XP_001130285	RQSILFSTDV	Human
94	DLG3-2	GRIN2B	2.85	NP_000825	EKLSSIESDV	Human
95	DLG3-2	NET1	2.91	NP_005854	SGGKRKETLV	Human
96	DLG3-2	KIF1B	2.91	NP_904325	NLKAGRETTV	Human
97	DLG3-2	DCUN1D1	2.93	NP_065691	QIAGTKSTTV	Human
98	DLG3-2	RASSF6	3.03	NP_803876	KLVIKTETTV	Human
99	DLG3-2	KCNA3	3.09	NP_002223	VNIKKIFTDV	Human
100	DLG3-2	C9orf166	3.10	NP_001073984	ARSRSISTDV	Human
101	DLG3-2	LOC642968	3.10	XP_943747	ARSRSISTDV	Human
102	DLG3-2	LOC643750	3.10	XP_932133	ARSRSISTDV	Human
103	DLG3-2	CRIPT	3.17	NP_054890	DTKNYKQTSV	Human
104	DLG3-2	CYSLTR2	3.29	NP_065110	SVWLRKETRV	Human
105	DLG3-2	ZNF599	3.29	NP_001007248	THHRKIHTRV	Human
106	DLG3-2	XKR7	3.37	NP_001011718	QELLEYETTV	Human
107	DLG3-2	MAS1	3.49	NP_002368	CNTVTVETVV	Human

	PDZ domain	Prioritized ligand	Interaction score	RefSeq ID	C terminal motif	Origin
108	DLG3-2	ERBB4	3.57	NP_001036064	PPYRHRNTVV	Human
109	DLG3-2	DUSP10	3.62	NP_653329	PKLMGVETVV	Human
110	DLG3-2	PBK	3.68	NP_060962	HIVEALETDV	Human
111	DLG3-2	CNKSR2	3.94	NP_055742	HTHSYIETHV	Human
112	DLG3-2	SLC45A4	3.98	XP_938889	QGPVETESVV	Human
113	DLG4-3	CYSLTR2	2.00	NP_065110	SVWLRKETRV	Human
114	DLG4-3	RALBP1	2.69	NP_006779	PSRDRKETSI	Human
115	DLG4-3	CRIPT	3.30	NP_054890	DTKNYKQTSV	Human
116	DLG4-3	ANKRD50	3.88	NP_065070	SFNYKKETPL	Human
117	DVL2-1	ALDH5A1	5.32	NP_733936	ELKYVCYGGL	Human
118	DVL2-1	LOC642727	5.67	XP_001126301	EIVNKLLGWI	Human
119	DVL2-1	VILL	5.67	NP_056957	RQEKKQLGFF	Human
120	DVL2-1	C14orf152	5.72	NP_612353	PPIFNVFGYL	Human
121	DVL2-1	UBE3A	5.84	NP_570854	ITYAKGFGML	Human
122	DVL2-1	PCDHGA12	6.10	NP_115265	LYQIFFLFFF	Human
123	DVL2-1	EMR4	6.30	NP_001073967	LSLINLLGIF	Human
124	DVL2-1	ARGFX	6.43	NP_001012677	TSNMVDLGFL	Human
125	DVL2-1	LOC730926	6.70	XP_001129902	NIRFRDFIIF	Human
126	DVL2-1	CAD	6.92	NP_004332	ALLATVLGRF	Human
127	ERBB2IP-1	CTNND2	5.52	NP_001323	HYPASPDSWV	Human
128	ERBB2IP-1	PKP4	5.52	NP_003619	QYPGSPDSWV	Human
129	ERBB2IP-1	ARVCF	5.94	NP_001661	AKPQPVDSWV	Human
130	HTRA1-1	YP_654474	2.10	YP_654474	QQLIDKCFIV	Viral
131	HTRA1-1	NP_149540	2.40	NP_149540	EDDDWKIDLF	Viral
132	HTRA1-1	NP_938294	3.38	NP_938294	NLRKDDIFLV	Viral
133	HTRA1-1	NP_734083	3.61	NP_734083	VNFINEIDHF	Viral
134	HTRA1-1	NP_047432	3.73	NP_047432	VTDFIKIFVV	Viral
135	HTRA1-1	NP_054056	3.73	NP_054056	VTDFIKIFVV	Viral
136	HTRA1-1	YP_758493	3.73	YP_758493	VTDFIKIFVV	Viral
137	HTRA1-1	YP_001110951	3.84	YP_001110951	RHVFDKIWEV	Viral
138	HTRA1-1	NP_703017	4.02	NP_703017	VTNFIKIFVV	Viral
139	HTRA1-1	GSTA1	4.21	NP_665683	LEEARKIFRF	Human
140	HTRA1-1	GSTA3	4.21	NP_000838	LEEARKIFRF	Human
141	HTRA1-1	GSTA5	4.21	NP_714543	LEEARKIFRF	Human
142	HTRA1-1	GSTA2	4.38	NP_000837	LEESRKIFRF	Human
143	HTRA1-1	SPAM1	4.40	NP_003108	DQGISRIGFF	Human
144	HTRA1-1	LYSMD1	4.50	NP_997716	RDQEDEIFKL	Human
145	HTRA1-1	GPR158	4.76	NP_065803	KEEIWDSFKV	Human
146	HTRA1-1	OR1N2	4.77	NP_001004457	LFVSGKTFFL	Human
147	HTRA1-1	LOC647169	4.79	XP_946882	LQEATKIFKV	Human
148	HTRA1-1	TAF11	4.91	NP_005634	NSKHKKIIFF	Human
149	HTRA1-1	FCHSD2	4.99	NP_055639	KIEDVEITLV	Human
150	HTRA2-1	NP_477645	2.88	NP_477645	NNYIFFFFLF	Viral
151	HTRA2-1	YP_025216	3.22	YP_025216	LKYLLFFIFF	Viral
152	HTRA2-1	LOC732241	3.22	XP_001132347	WLSAFLIILF	Human
153	HTRA2-1	HCG27	4.00	NP_859068	SPLIWKFFSL	Human
154	HTRA2-1	TMEM31	4.02	NP_872347	FIIVFILIFF	Human
155	HTRA2-1	LOC347475	4.37	XP_001128386	ILDHFTGDFF	Human
156	HTRA2-1	OR2M2	4.43	NP_001004688	KILALIMYIA	Human
157	HTRA2-1	LOC283902	4.52	XP_001134235	PFLGLVFILG	Human
158	HTRA2-1	OR5B12	4.54	NP_001004733	KAKASIGFIF	Human
159	HTRA2-1	STX5	4.58	NP_003155	FFIIFVVFLA	Human
160	HTRA2-1	OR5B3	4.72	NP_001005469	KAKLSVGWSV	Human
161	HTRA2-1	C11orf10	4.80	NP_055021	FLLLWVGIYV	Human
162	HTRA2-1	KBTBD4	4.80	NP_057590	LLTNLQFVLA	Human
163	HTRA2-1	TAS2R41	4.85	NP_795364	LLLARGFWVA	Human
164	HTRA2-1	NBPF3	4.88	XP_001129803	LHLVLQIWVI	Human

	PDZ domain	Prioritized ligand	Interaction score	RefSeq ID	C terminal motif	Origin
165	HTRA3-1	YP_425091	2.04	YP_425091	EPHDSQATWI	Viral
166	HTRA3-1	NP_037581	2.07	NP_037581	MAEKWFERWV	Viral
167	HTRA3-1	ZNF558	2.42	NP_653294	VHKRIHNRWI	Human
168	HTRA3-1	SELENBP1	2.71	NP_003935	GGDCSSDIWI	Human
169	HTRA3-1	MGC22014	2.73	XP_371501	KVTGPYSRWI	Human
170	HTRA3-1	MMP16	2.80	NP_005932	YCKRSMQEWV	Human
171	HTRA3-1	TRIM55	2.89	NP_149047	CLIFTLMDWI	Human
172	HTRA3-1	MMP15	2.97	NP_002419	YCKRSLQEWV	Human
173	INADL-2	CNTNAP4	1.72	NP_207837	VNENQKEYFF	Human
174	INADL-2	NRXN3	2.77	NP_004787	QKNKDREYYV	Human
175	INADL-3	NP_612295	5.23	NP_612295	SRNTVRFMDI	Viral
176	INADL-3	LOC646675	6.71	XP_934714	AEKEEWPLDI	Human
177	INADL-3	LOC650667	6.71	XP_944849	AEKEEWPLDI	Human
178	INADL-6	SLCO1C1	3.77	NP_059131	NYWPGKETQL	Human
179	INADL-6	SLC6A12	3.87	NP_003035	LIAGEKETHL	Human
180	LIN7A-1	CYSLTR2	5.31	NP_065110	SVWLRKETRV	Human
181	LIN7A-1	RAPGEF5	5.40	NP_036426	ELSHRIEPRV	Human
182	LRRC7-1	YP_294208	6.08	YP_294208	GVVSFIKTWL	Viral
183	LRRC7-1	CNKSR2	6.34	NP_055742	HTHSYIETHV	Human
184	LRRC7-1	ABCC4	7.00	NP_005836	STLTIFETAL	Human
185	LRRC7-1	TANC1	7.20	NP_203752	PKRSFIESNV	Human
186	LRRC7-1	CD109	7.46	NP_598000	KLLYFMELWL	Human
187	MAGI1-2	NP_039054	2.23	NP_039054	IRVRKSKTLL	Viral
188	MAGI1-2	NET1	2.56	NP_005854	SGGKRKETLV	Human
189	MAGI1-2	DSCAM	2.69	NP_001380	NPYAKSYTLV	Human
190	MAGI1-2	DSCAML1	2.94	NP_065744	GAYSKSYTLV	Human
191	MAGI1-4	ZMYM5	5.38	NP_001034739	HLHNARVLDV	Human
192	MAGI1-4	PARK2	5.39	NP_004553	VCMGDHWFDV	Human
193	MAGI3-2	YP_309009	2.14	YP_309009	IKEKIINTIV	Viral
194	MAGI3-2	YP_293816	2.67	YP_293816	NTENPFMTIV	Viral
195	MAGI3-2	NP_477767	2.68	NP_477767	FKKFFWVTRV	Viral
196	MAGI3-2	NP_477768	2.68	NP_477768	FKKFFWVTRV	Viral
197	MAGI3-2	NP_042054	2.70	NP_042054	DILNNDETIV	Viral
198	MAGI3-2	YP_249732	2.77	YP_249732	VKEKVINTIV	Viral
199	MAGI3-2	C3orf32	2.91	NP_057015	ERYCCGCTIV	Human
200	MAGI3-2	LOC401498	2.96	NP_997723	QGRWDHETIV	Human
201	MAGI3-3	PARK2	4.35	NP_004553	VCMGDHWFDV	Human
202	MAGI3-3	PRKG1	4.98	NP_006249	DDNSGWDIDF	Human
203	MAGI3-3	LOC646675	5.89	XP_934714	AEKEEWPLDI	Human
204	MAGI3-3	LOC650667	5.89	XP_944849	AEKEEWPLDI	Human
205	MAGI3-3	C10orf78	6.10	NP_660290	NRSEEEFIDV	Human
206	MLLT4-1	PCSK6	2.33	NP_612195	WWTIGWPWNV	Human
207	MLLT4-1	PVRL1	2.49	NP_002846	SFISKKEWYV	Human
208	MLLT4-1	KCTD7	2.58	NP_694578	PIYEFKITWW	Human
209	MLLT4-1	ZIC3	2.85	NP_003404	LPPNFNEWYV	Human
210	MLLT4-1	CNTNAP2	2.95	NP_054860	IDESKKEWLI	Human
211	MLLT4-1	GPR156	2.98	NP_694547	FKDDLKPTLV	Human
212	MPDZ-1	NP_478028	2.23	NP_478028	FCLAAFRFWI	Viral
213	MPDZ-1	PCDHA1	2.31	NP_113598	NVSPTFEFWL	Human
214	MPDZ-1	WWTR1	2.40	NP_056287	NKSEPFLTWL	Human
215	MPDZ-1	PCDHGA12	2.54	NP_115265	LYQIFFLFFF	Human
216	MPDZ-1	YAP1	2.63	NP_006097	LDKESFLTWL	Human
217	MPDZ-1	SPO11	2.68	NP_036576	PNKLKFGGWI	Human
218	MPDZ-1	PLEKHC1	2.71	NP_006823	MFYKLTSGWV	Human
219	MPDZ-1	LOC642727	2.88	XP_001126301	EIVNKLLGWI	Human
220	MPDZ-10	PFN2	2.47	NP_002619	LALYLRRSDV	Human
221	MPDZ-10	OR5H14	3.94	NP_001005514	FTKMFKRNDV	Human

	PDZ domain	Prioritized ligand	Interaction score	RefSeq ID	C terminal motif	Origin
222	MPDZ-12	LOC732425	3.99	XP_001133480	GQSILFSTDV	Human
223	MPDZ-12	ABR	4.07	NP_068781	RNTLYFSTDV	Human
224	MPDZ-12	LOC644165	4.21	XP_938794	RQSILFSTDV	Human
225	MPDZ-12	LOC731634	4.21	XP_001130285	RQSILFSTDV	Human
226	MPDZ-12	JOSD1	4.76	NP_055691	EAHQSWRTDV	Human
227	MPDZ-13	YP_529725	3.88	YP_529725	VIDVVTETKV	Viral
228	MPDZ-13	NP_963926	4.05	NP_963926	FFNWLKEERI	Viral
229	MPDZ-13	NP_963967	4.05	NP_963967	FFNWLKEERI	Viral
230	MPDZ-13	NP_039803	4.21	NP_039803	FFNWLREERI	Viral
231	MPDZ-13	CYSLTR2	4.23	NP_065110	SVWLRKETRV	Human
232	MPDZ-13	ASNSD1	4.24	NP_061921	NLSIEKETKL	Human
233	MPDZ-13	STARD13	4.72	NP_443083	LIAEGPETKI	Human
234	MPDZ-13	RPS6KA2	4.91	NP_001006933	GMKRLTSTRL	Human
235	MPDZ-2	YP_001029369	2.88	YP_001029369	CSWCLAFLWV	Viral
236	MPDZ-2	SEC22B	3.14	NP_004883	LIVYVRFWWL	Human
237	MPDZ-2	CD109	3.93	NP_598000	KLLYFMELWL	Human
238	MPDZ-2	BBS12	3.98	NP_689831	SQELTGFLFL	Human
239	MPDZ-3	NP_477645	2.39	NP_477645	NNYIFFFFLF	Viral
240	MPDZ-3	NP_149789	2.44	NP_149789	IVNHFNIFYL	Viral
241	MPDZ-3	NP_149673	2.87	NP_149673	NFIIFYIFKI	Viral
242	MPDZ-3	PCDHGA12	2.95	NP_115265	LYQIFFLFFF	Human
243	MPDZ-3	FAM124A	3.23	NP_659456	GDNDMEEFYI	Human
244	MPDZ-3	C12orf55	3.33	XP_001127217	DNLIRCLFYF	Human
245	MPDZ-3	ZNF66	3.36	XP_001132102	NFQSILFFFF	Human
246	MPDZ-3	FBXO32	3.38	NP_680482	PQDFINLFKF	Human
247	MPDZ-3	FLJ45557	3.41	NP_001034851	KHGRWKLWFL	Human
248	MPDZ-3	SULT4A1	3.44	NP_055166	KCDLTFDFYL	Human
249	MPDZ-3	MCM3	3.64	NP_002379	MVSEGIIFLI	Human
250	MPDZ-3	KIAA1328	3.64	NP_065827	NQILEDIFFI	Human
251	MPDZ-3	FBXO25	3.73	NP_904356	PQHFIDLFKF	Human
252	MPDZ-3	NAT1	3.75	XP_001127234	PKHGDRFFTI	Human
253	MPDZ-3	C6orf60	3.81	NP_078857	QEWFARYFTF	Human
254	MPDZ-3	TMEM76	3.82	XP_945851	LYRKKIFWKI	Human
255	MPDZ-3	GCNT2	3.84	NP_001482	ETAIQPSWYF	Human
256	MPDZ-3	SRD5A1	3.86	NP_001038	FRKIIIPFLF	Human
257	MPDZ-3	ATXN2L	3.89	NP_059867	DGRSFIFYYL	Human
258	MPDZ-3	CDR1	3.91	NP_004056	FWKTLIDWKI	Human
259	MPDZ-3	LYSMD1	3.92	NP_997716	RDQEDEIFKL	Human
260	MPDZ-4	PCDHGA12	5.99	NP_115265	LYQIFFLFFF	Human
261	MPDZ-4	VILL	6.15	NP_056957	RQEKKQLGFF	Human
262	MPDZ-4	TMEM31	6.91	NP_872347	FIIVFILIFF	Human
263	MPDZ-5	NP_477591	3.74	NP_477591	TCDEISGFFV	Viral
264	MPDZ-5	NP_048714	5.41	NP_048714	SGTNFSGDFV	Viral
265	MPDZ-5	NP_065045	5.48	NP_065045	YNKNFYIFFY	Viral
266	MPDZ-5	MIB2	5.52	NP_543151	PIRDRIQIFV	Human
267	MPDZ-5	TNFRSF10C	5.65	NP_003832	VLIVLLIVFV	Human
268	MPDZ-5	LOC391656	5.82	XP_373027	TGGKILGFFF	Human
269	MPDZ-5	LOC648068	5.82	XP_942213	TGGKILGFFF	Human
270	MPDZ-5	LOC728774	5.82	XP_001129390	TGGKILGFFF	Human
271	MPDZ-5	LOC730786	5.82	XP_001127371	TGGKILGFFF	Human
272	MPDZ-5	LOC732028	5.82	XP_001131640	TGGKILGFFF	Human
273	MPDZ-5	RPS15A	5.82	NP_001010	TGGKILGFFF	Human
274	MPDZ-5	MESP2	5.82	NP_001035047	LEGARLGIFY	Human
275	MPDZ-7	NP_149846	1.72	NP_149846	IFQIYKNTWF	Viral
276	MPDZ-7	WWTR1	1.97	NP_056287	NKSEPFLTWL	Human
277	MPDZ-7	YAP1	2.15	NP_006097	LDKESFLTWL	Human
278	MPDZ-7	FAM55B	2.24	NP_872301	ITSVQRHTWL	Human

	PDZ domain	Prioritized ligand	Interaction score	RefSeq ID	C terminal motif	Origin
279	MPDZ-7	C1orf190	2.37	NP_001013633	EQCQDDVTFL	Human
280	MPDZ-7	LOC730000	2.56	XP_001132025	NKLAVPRTWL	Human
281	MPDZ-7	LOC731055	2.56	XP_001128278	NKLAVPRTWL	Human
282	MPDZ-7	R3HDML	2.77	NP_848586	GLKSNKFTWF	Human
283	MPDZ-7	LOC728833	2.79	XP_001128582	GKKQLTWTFL	Human
284	MPDZ-7	NFX1	2.80	NP_667344	EVETSHWTFL	Human
285	MPDZ-7	KCTD7	2.89	NP_694578	PIYEFKITWW	Human
286	MPDZ-7	LOC647123	3.00	XP_001129691	HQXGHSHCWL	Human
287	MPDZ-9	GTF3C1	3.94	NP_001511	EVNWNKWIHL	Human
288	MPDZ-9	PARK2	4.59	NP_004553	VCMGDHWFDV	Human
289	MPDZ-9	YARS2	4.68	NP_001035526	NFYIIKWLQL	Human
290	MPP6-1	CADM2	0.95	NP_694854	NAEEKKEYFI	Human
291	MPP6-1	CADM1	1.11	NP_055148	NSEEKKEYFI	Human
292	MPP6-1	CADM3	1.23	NP_067012	GGDDKKEYFI	Human
293	MPP6-1	CNTNAP5	1.68	NP_570129	VSECKREYFI	Human
294	MPP6-1	LOC57228	1.80	NP_001026798	KGSEKEEYFI	Human
295	MPP6-1	GLULD1	1.83	NP_057655	ERNKFLEYFI	Human
296	MPP6-1	GYPC	2.39	NP_058131	GDSSRKEYFI	Human
297	MPP6-1	FLJ21511	2.49	NP_079363	FHMNTPKYFL	Human
298	MPP6-1	CNTNAP4	2.64	NP_207837	VNENQKEYFF	Human
299	MPP6-1	FLJ45557	2.64	NP_001034851	KHGRWKLWFL	Human
300	MPP6-1	AFAR3	2.96	NP_957704	FAHECPNYFI	Human
301	MPP6-1	LOC100037417	3.14	NP_001077862	NEEALFIYFI	Human
302	MPP6-1	PPP4C	3.22	NP_002711	SKKPVADYFL	Human
303	MPP6-1	ABCF3	3.54	NP_060828	QEQFRREGFL	Human
304	MPP6-1	LOC730134	3.60	XP_001132580	HCCFINQYYI	Human
305	MPP6-1	LOC732308	3.60	XP_001132712	HCCFINQYYI	Human
306	MPP6-1	LTB4R2	3.63	NP_062813	MEKDGPEWDL	Human
307	MPP6-1	C2orf32	3.77	NP_056278	LMWVNKESFL	Human
308	MPP6-1	NRXN1	3.83	NP_620072	KKNKDKEYYV	Human
309	MPP6-1	NRXN2	3.83	NP_620063	KKNKDKEYYV	Human
310	PARD3-3	NP_542524	1.52	NP_542524	KDIAEDLGWL	Viral
311	PARD3-3	NP_861613	1.52	NP_861613	KDIAEDLGWL	Viral
312	PARD3-3	POGZ	2.80	NP_997054	EEADLDLMEI	Human
313	PDLIM2-1	NP_041095	1.38	NP_041095	DCMCDRQDWL	Viral
314	PDLIM2-1	NP_041173	1.38	NP_041173	DCMCDRQDWL	Viral
315	PDLIM2-1	YP_068286	1.50	YP_068286	FPWKAEDPWL	Viral
316	PDLIM2-1	NP_542524	1.90	NP_542524	KDIAEDLGWL	Viral
317	PDLIM2-1	NP_861613	1.90	NP_861613	KDIAEDLGWL	Viral
318	PDLIM2-1	YP_001129474	2.11	YP_001129474	GNNNYNWPWL	Viral
319	PDLIM2-1	YP_401680	2.11	YP_401680	GNNNYNWPWL	Viral
320	PDLIM2-1	YP_293878	2.37	YP_293878	KGEAYPTGWL	Viral
321	PDLIM2-1	NP_064260	2.37	NP_064260	YHKELSRYWL	Viral
322	PDLIM2-1	YP_067976	2.40	YP_067976	GNKPYNWPWL	Viral
323	PDLIM2-1	C10orf65	2.43	NP_612422	RMDFTSNGWL	Human
324	PDLIM2-1	TRRAP	2.72	NP_003487	RMDPAWHPWL	Human
325	PDLIM2-1	PCDHA1	2.94	NP_113598	NVSPTFEFWL	Human
326	PDLIM2-1	LOC647123	3.00	XP_001129691	HQXGHSHCWL	Human
327	PDLIM4-1	MMAB	4.83	NP_443077	NDPSAESEGL	Human
328	PDZK1-1	NP_203189	3.72	NP_203189	EPHDVKSSRL	Viral
329	PDZK1-1	C10orf113	3.94	NP_001010896	SKKVLKMTFL	Human
330	PDZK1-1	GLRB	4.00	NP_000815	FNVIYWSIYL	Human
331	PDZK1-2	WHDC1L2	3.44	XP_001133241	IKEIIKVSKF	Human
332	PDZK1-2	CEP164	4.06	NP_055771	EHNRVKVYRF	Human
333	PDZK1-2	EIF1	4.71	NP_005792	KDDQLKVHGF	Human
334	PDZK1-2	EIF1B	4.71	NP_005866	KEEQLKVHGF	Human
335	PDZK1-2	LOC730144	4.71	XP_001132642	KDDQLKVHVF	Human

	PDZ domain	Prioritized ligand	Interaction score	RefSeq ID	C terminal motif	Origin
336	PDZK1-2	LOC731937	4.71	XP_001131332	KDDQLKVHVF	Human
337	PSCDBP-1	LOC730114	4.55	XP_001132496	DEDITLTRWL	Human
338	PSCDBP-1	LOC730899	4.55	XP_001127673	DEDITLTRWL	Human
339	PSCDBP-1	MTTP	4.66	NP_000244	QPDSTSSGWF	Human
340	PTPN13-2	FOXJ3	0.20	NP_055762	QDDFDWDSIV	Human
341	PTPN13-2	CRTAM	0.81	NP_062550	KHIQVPESIV	Human
342	PTPN13-4	NP_659715	2.05	NP_659715	IRVIFHMFFV	Viral
343	PTPN13-4	NP_115480	2.29	NP_115480	KRLFKDLFFV	Viral
344	PTPN13-4	NP_543049	2.29	NP_543049	KRLFKDLFFV	Viral
345	PTPN13-4	NP_740757	2.29	NP_740757	KRLFKDLFFV	Viral
346	PTPN13-4	NP_065006	2.31	NP_065006	VEHKKNIFSV	Viral
347	PTPN13-4	NP_477591	2.37	NP_477591	TCDEISGFFV	Viral
348	PTPN13-4	YP_717336	2.45	YP_717336	KNISYYIFSV	Viral
349	PTPN13-4	NP_077610	2.55	NP_077610	HRDDPVWFFV	Viral
350	PTPN13-4	NP_046151	2.64	NP_046151	KRLFRDLFFV	Viral
351	PTPN13-4	NP_955225	2.65	NP_955225	ETEDNDDVFV	Viral
352	PTPN13-4	NP_149914	2.75	NP_149914	DEKDNYDFYV	Viral
353	PTPN13-4	NP_694450	2.83	NP_694450	KRHKHKHWFF	Viral
354	PTPN13-4	NP_077524	2.83	NP_077524	DGQKVTIFSV	Viral
355	PTPN13-4	NP_891863	2.84	NP_891863	IKDVIRLYFV	Viral
356	PTPN13-4	YP_784209	2.84	YP_784209	EPTATYQFFV	Viral
357	PTPN13-4	YP_803550	2.90	YP_803550	KVIYDDHVFV	Viral
358	PTPN13-4	NP_065045	2.92	NP_065045	YNKNFYIFFY	Viral
359	PTPN13-4	NP_037870	2.95	NP_037870	FSHAQKLYFV	Viral
360	PTPN13-4	ZNF66	2.99	XP_001132102	NFQSILFFFF	Human
361	PTPN4-1	YP_227462	2.62	YP_227462	DYDYIKGTYV	Viral
362	PTPN4-1	NP_149846	2.84	NP_149846	IFQIYKNTWF	Viral
363	PTPN4-1	NP_050225	2.92	NP_050225	IMKQEKGTGV	Viral
364	PTPN4-1	FRAS1	3.09	NP_079350	HNNLQDGTEV	Human
365	PTPN4-1	KCTD7	3.14	NP_694578	PIYEFKITWW	Human
366	PTPN4-1	GSG1L	3.20	NP_653276	RQCWVLGHWV	Human
367	PTPN4-1	KCNH3	3.22	NP_036416	QWTQEEGTGV	Human
368	PTPN4-1	BAI1	3.32	NP_001693	QDIIDLQTEV	Human
369	PTPN4-1	LOC643965	3.32	XP_932312	DEDEQLCAWV	Human
370	PTPN4-1	LOC731292	3.35	XP_001130021	EDQHTQITKV	Human
371	PTPN4-1	PTEN	3.35	NP_000305	EDQHTQITKV	Human
372	PTPN4-1	KCNA3	3.37	NP_002223	VNIKKIFTDV	Human
373	PTPN4-1	KCTD14	3.45	NP_076419	NIYSFTFTWW	Human
374	PTPN4-1	BAI3	3.59	NP_001695	VQEGDFQTEV	Human
375	PTPN4-1	LOC729967	3.70	XP_001131915	ANDLDQGTAV	Human
376	PTPN4-1	LOC732175	3.70	XP_001132083	ANDLDQGTAV	Human
377	PTPN4-1	SYF2	3.70	NP_056299	KQNLERGTAV	Human
378	PTPN4-1	DLL1	3.75	NP_005609	KDECVIATEV	Human
379	PTPN4-1	FAT3	3.75	XP_931292	FVETQHQTQV	Human
380	PTPN4-1	PLAUR	3.75	NP_001005376	CCEEAQATHV	Human
381	PTPN4-1	SPAST	3.75	NP_055761	WNKDFGDTTV	Human
382	PTPN4-1	CRIPT	3.76	NP_054890	DTKNYKQTSV	Human
383	PTPN4-1	C11orf52	3.80	NP_542390	RYDSKNGTLV	Human
384	PTPN4-1	MMP16	3.80	NP_005932	YCKRSMQEWV	Human
385	PTPN4-1	SLC1A7	3.80	NP_006662	IQISELETNV	Human
386	PTPN4-1	CNKSR2	3.84	NP_055742	HTHSYIETHV	Human
387	PTPN4-1	DGKZ	3.84	NP_963290	IQREDQETAV	Human
388	PTPN4-1	GPR87	3.84	NP_076404	VRIYYDYTDV	Human
389	PTPN4-1	LOC401498	3.84	NP_997723	QGRWDHETIV	Human
390	PTPN4-1	ST14	3.84	NP_068813	RDWIKENTGV	Human
391	PTPN4-1	FRMD4B	3.87	XP_114303	HEDSKPGTLV	Human
392	PTPN4-1	C1orf183	3.87	NP_061972	GFDINTAVWV	Human

	PDZ domain	Prioritized ligand	Interaction score	RefSeq ID	C terminal motif	Origin
393	PTPN4-1	KIAA1919	3.90	NP_699200	PETRTKGTNV	Human
394	PTPN4-1	C3orf32	3.92	NP_057015	ERYCCGCTIV	Human
395	PTPN4-1	FCHSD2	3.92	NP_055639	KIEDVEITLV	Human
396	PTPN4-1	MMP24	3.92	NP_006681	YYKRPVQEWV	Human
397	PTPN4-1	ODF4	3.92	NP_694552	LDPEQKDTHV	Human
398	PTPN4-1	SEC14L3	3.92	NP_777635	QKYDKELTPV	Human
399	PTPN4-1	KIRREL	3.92	NP_060710	RFQQRMQTHV	Human
400	PTPN4-1	DGKI	3.93	NP_004708	IGHEDLETAV	Human
401	PTPN4-1	PBK	3.93	NP_060962	HIVEALETDV	Human
402	PTPN4-1	LOC285382	3.95	NP_001020437	EVIETIETTV	Human
403	PTPN4-1	BAI2	3.97	NP_001694	PPDGDFQTEV	Human
404	PTPN4-1	CXXC6	3.97	NP_085128	HVAGPYNHWV	Human
405	PTPN4-1	EXOC4	3.97	NP_068579	ATKDKKITTV	Human
406	PTPN4-1	MMP15	3.98	NP_002419	YCKRSLQEWV	Human
407	PTPN4-1	MRPS34	4.00	NP_076425	DKGRAKGTPV	Human
408	PTPN4-1	FOXI1	4.00	NP_658982	VLYPREGTEV	Human
409	SCRIB-1	NP_062444	5.51	NP_062444	TRLYYLYTHL	Viral
410	SCRIB-1	SLC6A12	5.68	NP_003035	LIAGEKETHL	Human
411	SCRIB-1	PARP3	5.88	NP_005476	RLRYLLEVHL	Human
412	SCRIB-2	CNKSR2	4.20	NP_055742	HTHSYIETHV	Human
413	SCRIB-2	CTNNB1	5.08	XP_001133675	NQLAWFDTDL	Human
414	SCRIB-3	GUCY1A2	4.76	NP_000846	GTMFLRETSL	Human
415	SCRIB-3	KIAA1240	4.91	XP_944000	RTVHMFETFL	Human
416	SCRIB-3	KCNA5	4.97	NP_002225	CLDTSRETDL	Human
417	SHANK3-1	SLC19A3	1.44	NP_079519	ESNIIMSTKL	Human
418	SHANK3-1	OR52E2	1.82	NP_001005164	KEEYLIHTRF	Human
419	SHANK3-1	LOC124446	1.96	NP_919256	RESHWSRTRL	Human
420	SLC9A3R2-2	NP_149846	1.37	NP_149846	IFQIYKNTWF	Viral
421	SLC9A3R2-2	YP_956837	1.80	YP_956837	VQKPRCSTWL	Viral
422	SLC9A3R2-2	GUCY2C	2.34	NP_004954	NTTDKESTYF	Human
423	SLC9A3R2-2	ABCC2	2.47	NP_000383	GIENVNSTKF	Human
424	SLC9A3R2-2	PODXL	2.52	NP_005388	DLDEEEDTHL	Human
425	SLC9A3R2-2	CFTR	2.56	NP_000483	TEEEVQDTRL	Human
426	SLC9A3R2-2	CROT	2.58	NP_066974	MIQLMNSTHL	Human
427	SLC9A3R2-2	SLC19A3	2.70	NP_079519	ESNIIMSTKL	Human
428	SLC9A3R2-2	TBC1D10A	2.81	NP_114143	TSQESEDTYL	Human
429	SLC9A3R2-2	PODXL2	2.83	NP_056535	SDVFEEDTHL	Human
430	SNTA1-1	NP_048733	2.21	NP_048733	GNEDKIETRV	Viral
431	SNTA1-1	NP_477767	2.47	NP_477767	FKKFFWVTRV	Viral
432	SNTA1-1	NP_477768	2.47	NP_477768	FKKFFWVTRV	Viral
433	SNTA1-1	GUCY1A2	2.53	NP_000846	GTMFLRETSL	Human
434	SNTA1-1	KIF1B	2.72	NP_904325	NLKAGRETTV	Human
435	SNTA1-1	TRPV3	2.99	NP_659505	EVEEFPETSV	Human
436	SNTA1-1	GNG4	2.99	NP_004476	REKKFFCTIL	Human
437	TIAM1-1	YP_142666	5.50	YP_142666	EKKIVFLENF	Viral
438	TIAM1-1	PCDHGA12	6.63	NP_115265	LYQIFFLFFF	Human
439	TIAM1-1	SLC39A6	6.74	NP_036451	EHKIVFRINF	Human
440	TIAM2-1	CYSLTR2	2.99	NP_065110	SVWLRKETRV	Human
441	TIAM2-1	CRIPT	3.04	NP_054890	DTKNYKQTSV	Human
442	TJP1-1	YP_758316	4.00	YP_758316	DGGWILTTFL	Viral
443	TJP1-1	YP_294208	5.06	YP_294208	GVVSFIKTWL	Viral
444	TJP1-1	NP_042214	5.32	NP_042214	MNPYSFQTQV	Viral
445	TJP1-1	ZPBP	5.32	NP_008940	SLVYGAKTCL	Human
446	TJP1-1	CLDN14	5.51	NP_652763	HSGYRLNDYV	Human
447	TJP1-1	KALRN	5.90	NP_003938	GPGDPFSTYV	Human
448	TJP1-3	NP_037581	5.78	NP_037581	MAEKWFERWV	Viral
449	TJP1-3	LOC730114	5.87	XP_001132496	DEDITLTRWL	Human

	PDZ domain	Prioritized ligand	Interaction score	RefSeq ID	C terminal motif	Origin
450	TJP1-3	LOC730899	5.87	XP_001127673	DEDITLTRWL	Human
451	TJP2-3	LOC730114	4.80	XP_001132496	DEDITLTRWL	Human
452	TJP2-3	LOC730899	4.80	XP_001127673	DEDITLTRWL	Human
453	TJP2-3	LOC732156	5.56	XP_001132029	HSKCFIYDYI	Human
454	TJP2-3	FBXO33	5.88	NP_976046	REMQSFSEDI	Human
