# Supplementary material for: A Specificity Map for the PDZ Domain Family
Source: PLoS Biol. 2008 Sep 30;6(9):e239. doi: 10.1371/journal.pbio.0060239 (PMC2553845; doi:10.1371/journal.pbio.0060239)
Supplement: Table S6 — PDZ domains are listed in numerical order, and for each, the 50 ligands with the best prediction score are listed in ascending order by interaction score. Lower interaction scores are better. Known ligands reported in PDZBase for DLG1 or the other DLG homologs (DLG2, −3, and −4) are highlighted in yellow or blue, respectively. (41 KB PDF) [file pbio.0060239.st006.pdf]

Table S6. Prioritized endogenous ligands for the PDZ domains of DLG1.

PDZ domains are listed in numerical order and, for each, the 50 best predicted ligands are listed in ascending order by interaction score. Lower interaction scores are better. Known ligands reported in PDZBase for DLG1 or the other DLG homologs (DLG2, 3 and 4) are highlighted in yellow or blue, respectively.

	PDZ domain	Prioritized ligand	Interaction score	RefSeq ID	C terminal motif
1	DLG1-1	PBK	2.25	NP_060962	HIVEALETDV
2	DLG1-1	ARHGAP6	2.41	NP_038286	NPDALPETLV
3	DLG1-1	NET1	2.45	NP_005854	SGGKRKETLV
4	DLG1-1	KCNA4	2.51	NP_002224	SNAKAVETDV
5	DLG1-1	ARHGEF16	2.64	NP_055263	MERLRVETDV
6	DLG1-1	ABR	2.86	NP_068781	RNTLYFSTDV
7	DLG1-1	LOC732425	2.86	XP_001133480	GQSILFSTDV
8	DLG1-1	TMEM16J	3.00	NP_001012302	SIFSARSTDV
9	DLG1-1	LOC731634	3.04	XP_001130285	RQSILFSTDV
10	DLG1-1	ATP2B4	3.22	NP_001675	SSLQSLETSV
11	DLG1-1	C1orf76	3.37	NP_775780	TNPRAISTDV
12	DLG1-1	CNKSR2	3.56	NP_055742	HTHSYIETHV
13	DLG1-1	LOC401498	3.56	NP_997723	QGRWDHETIV
14	DLG1-1	DGKZ	3.56	NP_963290	IQREDQETAV
15	DLG1-1	XKR7	3.62	NP_001011718	QELLEYETTV
16	DLG1-1	FCHSD2	3.62	NP_055639	KIEDVEITLV
17	DLG1-1	KCNA3	3.72	NP_002223	VNIKKIFTDV
18	DLG1-1	LOC285382	3.73	NP_001020437	EVIETIETTV
19	DLG1-1	SLC1A7	3.74	NP_006662	IQISELETNV
20	DLG1-1	KIF1B	3.83	NP_904325	NLKAGRETTV
21	DLG1-1	JOSD1	3.87	NP_055691	EAHQSWRTDV
22	DLG1-1	TRPV3	3.91	NP_659505	EVEEFPETSV
23	DLG1-1	DGKI	3.91	NP_004708	IGHEDLETAV
24	DLG1-1	GPR123	3.99	NP_001077378	TGPWKNETTV
25	DLG1-1	ADRA1D	4.03	NP_000669	DYSNLRETDI
26	DLG1-1	LOC729755	4.05	XP_001131212	KEKQLEEKMV
27	DLG1-1	SLCO6A1	4.05	NP_775759	KVKKKEETDL
28	DLG1-1	C9orf166	4.07	NP_001073984	ARSRSISTDV
29	DLG1-1	MARCH2	4.08	NP_057580	LKKVAEETPV
30	DLG1-1	UBE2T	4.10	NP_054895	GIEKKFHPDV
31	DLG1-1	TGFA	4.12	NP_003227	TACCHSETVV
32	DLG1-1	GPR156	4.14	NP_694547	FKDDLKPTLV
33	DLG1-1	GPR87	4.16	NP_076404	VRIYYDYTDV
34	DLG1-1	GPR125	4.17	XP_001131071	TGLWKHETTV
35	DLG1-1	RASSF6	4.17	NP_803876	KLVIKTETTV
36	DLG1-1	FZD1	4.17	NP_003496	TNSKQGETTV
37	DLG1-1	MARCH3	4.21	NP_848545	VKRNSKETVV
38	DLG1-1	DSCAM	4.31	NP_001380	NPYAKSYTLV
39	DLG1-1	LRRC3B	4.33	NP_443185	DEPDDISTVV
40	DLG1-1	MAS1	4.33	NP_002368	CNTVTVETVV
41	DLG1-1	KALRN	4.36	NP_003938	GPGDPFSTYV
42	DLG1-1	FRMPD4	4.38	NP_055543	RLPKIKETTV
43	DLG1-1	FRMD4B	4.40	XP_114303	HEDSKPGTLV
44	DLG1-1	FZD4	4.43	NP_036325	KPGKGSETVV

	PDZ domain	Prioritized ligand	Interaction score	RefSeq ID	C terminal motif
45	DLG1-1	GRIN2A	4.45	NP_000824	KKMPSIESDV
46	DLG1-1	SMYD5	4.46	NP_006053	AELGDEMTDV
47	DLG1-1	KCNA2	4.46	NP_004965	VNITKMLTDV
48	DLG1-1	CRHR1	4.46	XP_001128344	FHSIKQSTAV
49	DLG1-1	ST14	4.49	NP_068813	RDWIKENTGV
50	DLG1-1	CCRL2	4.50	NP_003956	REEPDHSTEV
1	DLG1-2	LOC401498	1.97	NP_997723	QGRWDHETIV
2	DLG1-2	TMEM16J	2.06	NP_001012302	SIFSARSTDV
3	DLG1-2	NET1	2.21	NP_001040625	SGGKRKETLV
4	DLG1-2	KCNA4	2.33	NP_002224	SNAKAVETDV
5	DLG1-2	PBK	2.41	NP_060962	HIVEALETDV
6	DLG1-2	MARCH3	2.41	XP_001127871	VKRNSKETVV
7	DLG1-2	CRIPT	2.46	NP_054890	DTKNYKQTSV
8	DLG1-2	PLEKHA2	2.50	XP_945579	DDENIRTSDV
9	DLG1-2	ADRA1D	2.55	NP_000669	DYSNLRETDI
10	DLG1-2	KIF1B	2.59	NP_904325	NLKAGRETTV
11	DLG1-2	ARHGEF16	2.64	NP_055263	MERLRVETDV
12	DLG1-2	ATP2B4	2.66	NP_001675	SSLQSLETSV
13	DLG1-2	ERBB4	2.68	NP_005226	PPYRHRNTVV
14	DLG1-2	KCNA5	2.69	NP_002225	CLDTSRETDL
15	DLG1-2	GRIN2A	2.76	NP_000824	KKMPSIESDV
16	DLG1-2	DGKZ	2.79	NP_963290	IQREDQETAV
17	DLG1-2	LOC285382	2.82	NP_001020437	EVIETIETTV
18	DLG1-2	C1orf76	2.86	NP_775780	TNPRAISTDV
19	DLG1-2	PLAC8L1	2.92	NP_001025040	AVPMTKDTLV
20	DLG1-2	FRMPD4	2.94	NP_055543	RLPKIKETTV
21	DLG1-2	LRRC3B	2.94	NP_443185	DEPDDISTVV
22	DLG1-2	LOC732425	2.97	XP_001133480	GQSILFSTDV
23	DLG1-2	ABR	2.99	NP_068781	RNTLYFSTDV
24	DLG1-2	FAT3	3.00	XP_941631	FVETQHQTQV
25	DLG1-2	FAM55B	3.01	NP_872301	ITSVQRHTWL
26	DLG1-2	SLCO6A1	3.02	NP_775759	KVKKKEETDL
27	DLG1-2	ABCA1	3.03	NP_005493	QDEKVKESYV
28	DLG1-2	XKR7	3.04	NP_001011718	QELLEYETTV
29	DLG1-2	LOC731231	3.13	XP_001128895	SPDRDRESIV
30	DLG1-2	LOC731634	3.14	XP_001130285	RQSILFSTDV
31	DLG1-2	GPR125	3.17	XP_001131071	TGLWKHETTV
32	DLG1-2	ARHGAP6	3.17	NP_038286	NPDALPETLV
33	DLG1-2	BAI3	3.18	NP_001695	VQEGDFQTEV
34	DLG1-2	TRPV3	3.20	NP_659505	EVEEFPETSV
35	DLG1-2	ZNF599	3.21	NP_001007248	THHRKIHTRV
36	DLG1-2	CYSLTR2	3.27	NP_065110	SVWLRKETRV
37	DLG1-2	MAS1	3.27	NP_002368	CNTVTVETVV
38	DLG1-2	DCUN1D1	3.28	NP_065691	QIAGTKSTTV
39	DLG1-2	GRIN2B	3.31	NP_000825	EKLSSIESDV
40	DLG1-2	CCRL2	3.36	NP_003956	REEPDHSTEV
41	DLG1-2	SLCO1C1	3.39	NP_059131	NYWPGKETQL
42	DLG1-2	BAI1	3.40	XP_001130169	QDIIDLQTEV
43	DLG1-2	DUSP10	3.40	NP_653329	PKLMGVETVV
44	DLG1-2	CRHR1	3.41	XP_001128344	FHSIKQSTAV
45	DLG1-2	DGKI	3.42	NP_004708	IGHEDLETAV
46	DLG1-2	LOC642968	3.42	XP_943747	ARSRSISTDV

	PDZ domain	Prioritized ligand	Interaction score	RefSeq ID	C terminal motif
47	DLG1-2	GPR19	3.46	NP_006134	INSNPPNTFV
48	DLG1-2	BAI2	3.46	NP_001694	PPDGDFQTEV
49	DLG1-2	CACNG2	3.48	NP_006069	NTANRRTTPV
50	DLG1-2	TGFA	3.48	NP_003227	TACCHSETVV
1	DLG1-3	CRIPT	1.99	NP_054890	DTKNYKQTSV
2	DLG1-3	CYSLTR2	3.43	NP_065110	SVWLRKETRV
3	DLG1-3	KIF1B	3.50	NP_904325	NLKAGRETTV
4	DLG1-3	GUCY1A2	4.43	NP_000846	GTMFLRETSL
5	DLG1-3	TRPV3	4.56	NP_659505	EVEEFPETSV
6	DLG1-3	FRMPD4	4.69	NP_055543	RLPKIKETTV
7	DLG1-3	CIT	5.12	NP_009105	VNKVWDQSSV
8	DLG1-3	NET1	5.18	NP_005854	SGGKRKETLV
9	DLG1-3	SLCO1C1	5.24	NP_059131	NYWPGKETQL
10	DLG1-3	RALBP1	5.38	NP_006779	PSRDRKETSI
11	DLG1-3	XKR7	5.40	NP_001011718	QELLEYETTV
12	DLG1-3	FZD2	5.47	NP_001457	TNSRHGETTV
13	DLG1-3	GRWD1	5.54	NP_113673	GFTIFRTISV
14	DLG1-3	ERBB4	5.63	NP_005226	PPYRHRNTVV
15	DLG1-3	LOC647631	5.68	XP_948002	TPGPWINTSL
16	DLG1-3	CNKSR2	5.75	NP_055742	HTHSYIETHV
17	DLG1-3	NETO1	5.78	NP_694821	HESEYNTTRV
18	DLG1-3	CACNG2	5.78	NP_006069	NTANRRTTPV
19	DLG1-3	LRRC1	5.79	NP_060684	HAIDRVTTSV
20	DLG1-3	XKR4	5.81	NP_443130	QERLEYETTL
21	DLG1-3	CACNG3	5.86	NP_006530	NPANRRTTPV
22	DLG1-3	RSBN1	5.89	NP_060834	NLQEHSTTSV
23	DLG1-3	ARHGEF7	6.00	NP_003890	NDPAWDETNL
24	DLG1-3	WDR54	6.00	NP_115494	LAEIRRFSSV
25	DLG1-3	KLHDC7B	6.03	NP_612442	PPEDRLQTSL
26	DLG1-3	ASNSD1	6.04	NP_061921	NLSIEKETKL
27	DLG1-3	DHRS2	6.06	NP_005785	IAVAGYSTRL
28	DLG1-3	PRR7	6.09	NP_085044	IPLFGRTTAV
29	DLG1-3	TACSTD2	6.09	NP_002344	LGELRKEPSL
30	DLG1-3	LOC727829	6.10	XP_001125907	PRPPRRSSSL
31	DLG1-3	SLC16A3	6.13	NP_001035887	EVVHTPETSV
32	DLG1-3	CACNG8	6.15	NP_114101	NTLNRKTTPV
33	DLG1-3	CACNG4	6.15	NP_055220	SMLNRRTTPV
34	DLG1-3	ATP2B4	6.17	NP_001675	SSLQSLETSV
35	DLG1-3	OR1S2	6.17	NP_001004459	KLINRKISSL
36	DLG1-3	ACVR2A	6.21	NP_001607	VDFPPKESSL
37	DLG1-3	ATP2B2	6.29	NP_001001331	SPIHSLETSL
38	DLG1-3	RASSF6	6.31	NP_803876	KLVIKTETTV
39	DLG1-3	SLC4A7	6.33	NP_003606	KKYVDAETSL
40	DLG1-3	NEDD9	6.33	NP_892011	LVRASKQTSL
41	DLG1-3	SLC17A3	6.34	NP_006623	WAKERKLTRL
42	DLG1-3	VANGL2	6.34	NP_065068	VMRLQSETSV
43	DLG1-3	KCNB2	6.36	NP_004761	GYCPTRETSM
44	DLG1-3	DCUN1D1	6.37	NP_065691	QIAGTKSTTV
45	DLG1-3	C3AR1	6.41	NP_004045	VISERNSTTV
46	DLG1-3	XKR6	6.42	NP_775954	YELLQYESSL
47	DLG1-3	MUT	6.46	NP_000246	KCLEKKQQSV
48	DLG1-3	VANGL1	6.52	NP_620409	VLRLQSETSV

	PDZ domain	Prioritized ligand	Interaction score	RefSeq ID	C terminal motif
49	DLG1-3	MARCH3	6.53	XP_001127871	VKRNSKETVV
50	DLG1-3	NLGN1	6.53	NP_055747	HPHSHSTTRV
