# Supplementary material for: A Specificity Map for the PDZ Domain Family
Source: PLoS Biol. 2008 Sep 30;6(9):e239. doi: 10.1371/journal.pbio.0060239 (PMC2553845; doi:10.1371/journal.pbio.0060239)
Supplement: Table S7 — Known ligands reported in PDZBase are ordered in ascending order of interaction score for the highest scoring PDZ domain (Table S6). Lower interaction scores are better. NA denotes ligands that were not predicted by the scoring algorithm. (24 KB PDF) [file pbio.0060239.st007.pdf]

Table S7. Known ligands for the PDZ domains of DLG1.

Known ligands reported in PDZBase are ordered in ascending order of interaction score for the highest scoring PDZ domain (Supplementary Table S6). Lower interaction scores are better. NA denotes ligands that were not predicted by the scoring algorithm.

	**Prioritized ligand**	**Interaction score**	**RefSeq ID**	**C terminal motif**
1	PBK	2.25	NP_060962	HIVEALETDV
2	ATP2B4	2.66	NP_001675	SSLQSLETSV
3	Grin2a	2.76	NP_000824	KKMPSIESDV
4	Grin2b	3.31	NP_000825	EKLSSIESDV
5	Frizzled-2	4.07	NP_001457	TNSRHGETTV
6	Frizzled-1	4.17	NP_003496	TNSKQGETTV
7	Frizzled-4	4.43	NP_036325	KPGKGSETVV
8	ATP2B2	6.29	NP_001001331	SPLHSLETSL
9	Frizzled-7	NA	NP_003498	SHSSKGETAV
10	ADAM17	NA	NP_064702	SRVDSKETEC
11	Kir2.2	NA	P52188	ERPYRRESEI
